# Enhanced Removal of Ultratrace Levels of Gold from Wastewater Using Sulfur-Rich Covalent Organic Frameworks

**DOI:** 10.1021/acsami.4c03685

**Published:** 2024-06-01

**Authors:** Salma Abubakar, Gobinda Das, Thirumurugan Prakasam, Asmaa Jrad, Felipe Gándara, Sabu Varghese, Thomas Delclos, Mark A. Olson, Ali Trabolsi

**Affiliations:** † Science Division, New York University Abu Dhabi, Saadiyat Island, 129188 Abu Dhabi, United Arab Emirates; ‡ Water Research Centre, New York University Abu Dhabi, Saadiyat Island, 129118 Abu Dhabi, United Arab Emirates; § Materials Science Institute of Madrid—CSIC, Sor Juana Inés de la Cruz 3, 28049 Madrid, Spain; ∥ CTP, New York University Abu Dhabi, Saadiyat Island, 129188 Abu Dhabi, United Arab Emirates; ⊥ Materials and Surface Core Laboratories, Khalifa University of Science and Technology, 127788 Abu Dhabi, United Arab Emirates; # Department of Physical and Environmental Sciences, Texas A&M University Corpus Christi, 6300 Ocean Drive, Corpus Christi, Texas 78412 United States

**Keywords:** covalent organic framework, thioanisole groups, gold capture, selective adsorption, ppb levels, wastewater, saline conditions

## Abstract

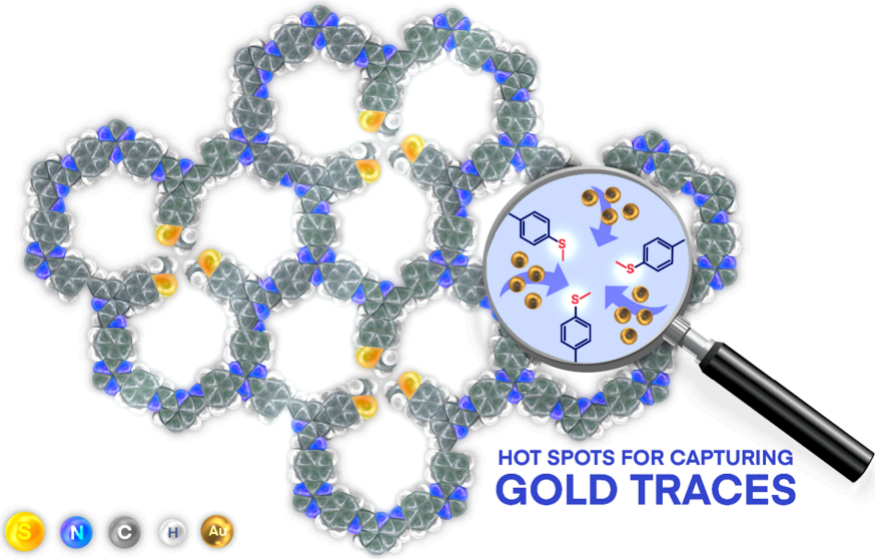

In view of the increasing global demand and consumption of gold, there is a growing need and effort to extract gold from alternative sources besides conventional mining, e.g., from water. This drive is mainly due to the potential benefits for the economy and the environment as these sources contain large quantities of the precious metal that can be utilized. Wastewater is one of these valuable sources in which the gold concentration can be in the ppb range. However, the effective selective recovery and recycling of ultratrace amounts of this metal remain a challenge. In this article, we describe the development of a covalent imine-based organic framework with pores containing thioanisole functional groups (TTASDFPs) formed by the condensation of a triazine-based triamine and an aromatic dialdehyde. The sulfur-functionalized pores served as effective chelating agents to bind Au^3+^ ions, as evidenced by the uptake of more than 99% of the 9 ppm Au^3+^ solution within 2 min. This is relatively fast kinetics compared with other adsorbents reported for gold adsorption. TTASDFP also showed a high removal capacity of 245 mg·g^–1^ and a clear selectivity toward gold ions. More importantly, the material can capture gold at concentrations as low as 1 ppb.

## 1 Introduction

As one of the rare precious metals, gold is of great importance for various industries and everyday applications, including jewelry manufacturing, the electronics industry, and biomedical and catalytic applications.^1,2^ Gold is especially essential for electronics manufacturing. For example, the gold content in electronic devices can exceed 200 g per ton.^3^ The extensive consumption of gold is mainly because of the metal’s unique physical and chemical properties, such as its excellent malleability, corrosion resistance, and high electrical conductivity.^4^ Due to the massive demand for gold, about 190 × 10^3^ tons have been mined worldwide to date, according to the World Gold Council.^5^ However, large-scale gold mining is a grueling process that can cause landscape degradation and soil and water contamination.^6^ In addition, acute exposure to gold can cause potential health problems in minors, including liver and kidney damage.^7,8^ These factors make conventional mining a challenging method of gold extraction, especially given the dwindling natural supply of the metal.^9^ On the other hand, large-scale industrial production of gold-containing products has resulted in significant amounts of gold being released into the environment as electronic waste (e-waste), which is considered a valuable source of scrap gold.^10^ In addition to ores and e-waste, gold is also largely found in other secondary sources, such as wastewater and seawater, in which the gold content is diluted to concentrations below 10 μg·L^–1^ or 10 ng·L^–1^.^11,12^ However, these sources remain largely untapped as the effective, selective, and permanent recovery of such trace metals remain a difficult task that requires active research.

There are several methods for gold recovery from secondary sources, including pyrometallurgy,^13,14^ hydrometallurgy,^15,16^ ion exchange resins,^17,18^ bio-oxidation,^19,20^ and cementation.^21,22^ However, many of these techniques are limited by drawbacks such as energy demand, operation cost, and the generation of hazardous substances.^23^ On the other hand, adsorption has attracted considerable attention due to its sustainability, ease of operation, and low cost compared to other methods.^24^ Various conventional adsorbents have been explored for gold recovery, such as activated carbon,^25,26^ biomass materials,^27,28^ organic polymers,^29,30^ and metal–organic frameworks (MOFs).^31,32^ Although these materials have good gold capture ability, some of them suffer from poor adsorption capacity, slow removal kinetics, or poor selectivity.^33,34^ Therefore, it is important to develop materials with properties that counteract these drawbacks to enable improved gold recovery even at low concentrations.

Covalent organic frameworks (COFs) are a class of covalent porous crystalline polymers composed of lightweight elements and known for their highly ordered structures and permeant porosity.^35^ COFs are also structurally predesignable by controlling the geometry and the chemical composition of the organic building blocks during the topology-directed network growth.^36^ With their advantageous properties of synthetic tunability, high surface area, and structural stability, COFs have been used in various applications, including gas capture,^37^ sensing,^38^ pollutant removal,^39,40^ and catalysis.^41^ Moreover, due to these properties, COFs have been previously employed for the removal of Au^3+^ from aqueous media, with several reported examples showing promising performances compared to conventional adsorbents.^42−45^ However, their gold selectivity and removal efficiency can be further enhanced via functionalizing with sulfur-based groups, such as thiols and thioethers, which are known for their strong binding to gold.^46,47^ Moreover, it was established the strength of gold–sulfur bond exceeds that of gold–nitrogen and gold–oxygen bonds.^48,49^ The strong binding is primarily driven by the soft acid–soft base interaction between gold and sulfur atoms, respectively.^50,51^ Moreover, it is reported that the strong Au–S affinity endowed the bond high stability in water, organic media, and air, which makes our S-based moieties great for gold recovery from solutions.^34^ Despite their premise as gold adsorbents, most sulfur-based COFs have been mainly targeted for Au recovery from ppm-level-containing solutions (Supporting Information Table S1).^52−55^ Therefore, there is a need to design COFs that can effectively remove gold trace levels close to those found in wastewater. One strategy is exploiting both the principle of hard and soft acid–base (HSAB), and the well-defined structures and porosity of COFs, in addition to manipulating pore environments by introducing pendant sulfur groups to the pore walls.^56^ Integrating gold binding groups to the pore surface maximizes the exposure of gold ion traces that diffuse to the sulfur-rich surfaces across the COF’s extended network.^57^ The premise of this strategy has been highlighted in a previous report on the sulfur-functionalized TTB-COF, which displayed clear gold sensing properties.^52^

**Scheme 1:**
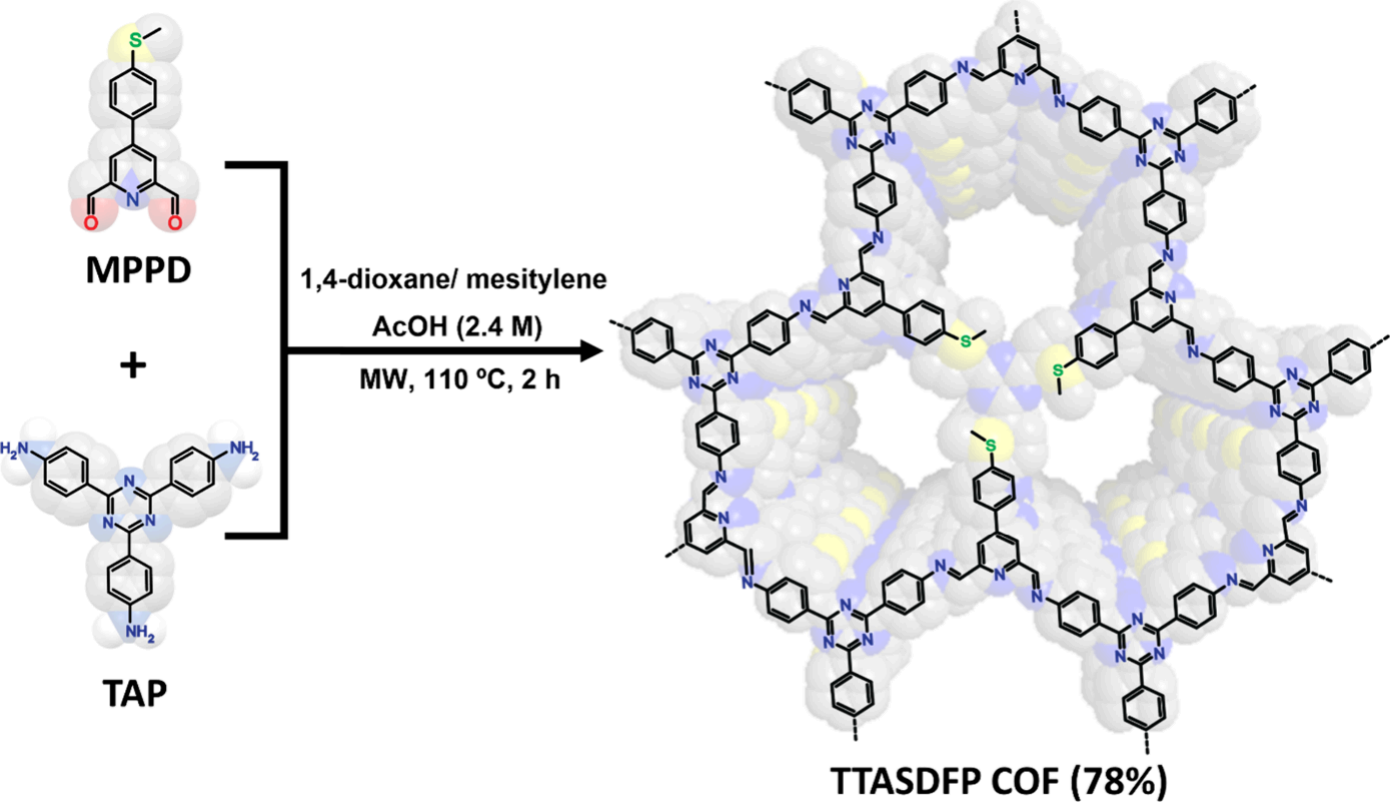
Synthetic Scheme and Chemical Structure of TTASDFP COF Which Was Obtained under Microwave (MW) Irradiation, with a Synthetic Yield of 78% The sulfur atoms are colored green for clarity.

Here, we report on an imine-based COF with thioanisole-rich pores (TTASDFP COF) through the condensation of 2,4,6-tris(4-aminophenyl)-1,3,5-triazine (TAP) and 4-(4-(methylthio)phenyl)pyridine-2,6-dicarbaldehyde (MPPD) (Scheme 1). In this study, we take advantage of the closely and periodically arranged sulfur atoms that are located at approximately 7.3 Å from each other, to create well-defined binding sites that effectively capture Au^3+^ at the ppm and ppb levels (Figure S1). The results show that TTASDFP exhibits the rapid sorption of gold ions with a high uptake capacity. The COF was also able to selectively remove gold ions in the presence of other metal ions. Moreover, the material successfully captured gold ions at levels below 16 ppb, even in the presence of high concentrations of sodium chloride and copper ions.

## 2 Experimental Section

### 2.1 Synthesis of TTASDFP

TTASDFP was prepared by the condensation of 4-(4-(methylthio)phenyl)pyridine-2,6-dicarbaldehyde (MPPD) with 2,4,6-tris(4-aminophenyl)-1,3,5-triazine (TAP) to form imine bonds. The integration of MPPD allowed for the enrichment of the COF pores with thiol groups that serve as hotspots for gold binding. The material was synthesized by microwave irradiation for 2 h, resulting in a powder product with a yield of 78%. After purification, the structural and morphological properties of TTASDFP were analyzed before its suitability for capturing gold ions from aqueous media was investigated.

## 3 Results and Discussion

### 3.1 Characterization of TTASDFP

The Fourier transform infrared (FT-IR) spectrum of TTASDFP showed the disappearance of the signals corresponding to the carbonyl C=O (1702 cm^–1^) and N–H bonds of the primary amines (3308–3413 cm^–1^) of MPPD and TAP precursors, respectively, indicating a successful condensation reaction (Figure S2). Moreover, TTASDFP showed a signal at ∼1601 cm^–1^, which can be assigned to the -C=N stretching of the imine bond. The presence of the imine bond was further confirmed by solid-state ^13^C cross-polarization magic-angle spinning (CP/MAS) NMR spectroscopy (Figure S3), where the carbon peak originating from the imine bond was observed at ∼160 ppm.^58^ Moreover, an additional C=N signal was observed at ∼171 ppm, corresponding to the triazine cores in the structure.^59^ An additional peak was also obtained at ∼15 ppm, originating from the alkyl carbon of the thioanisole.^60^ This confirmed the presence of the gold-binding moieties in TTASDFP’s structure.

**Figure 1:**
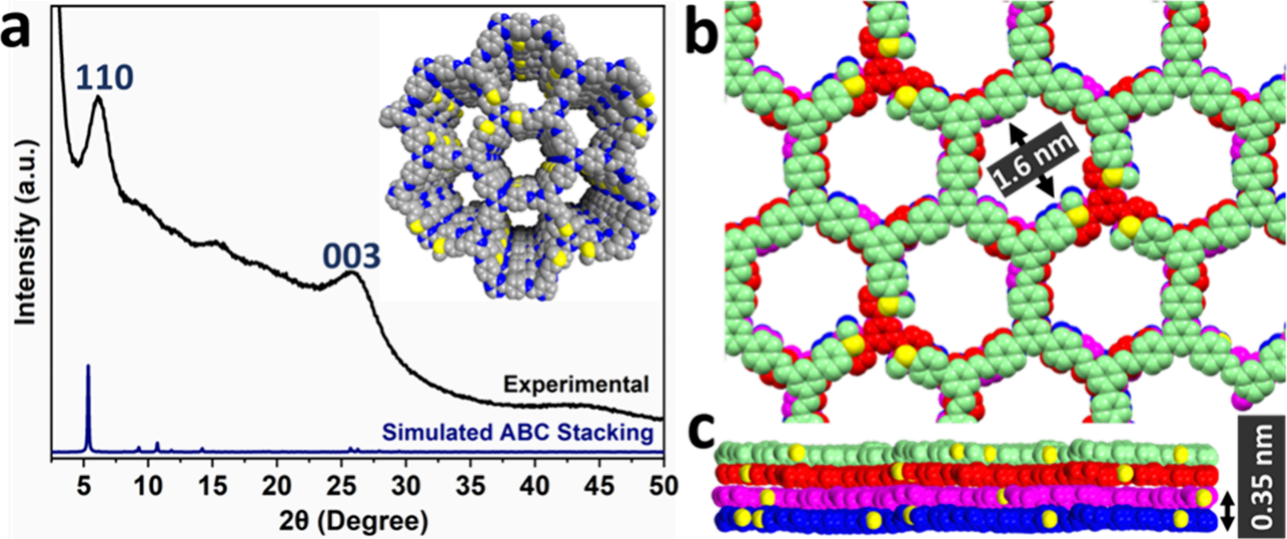
(a) Stacked experimental powder XRD diffraction pattern of TTASDFP (black line) and the simulated pattern from the ABC stacking (blue line). (b, c) Top and side views of TTASDFP’s simulated structure in the space-filling mode, in which the layers are in different colors and sulfur atoms are colored in yellow.

The ordered structure of TTASDFP was confirmed by powder X-ray diffraction (PXRD). The diffraction pattern of the material was observed in the low-angle region, with the main peaks occurring at 2θ = 6.1 and 25.8° (Figure 1a). Based on the principles of reticular chemistry, an optimal structural model of TTASDFP was constructed and geometrically optimized using universal force field energy minimizations (Figure. 1b). The structural model consisted of a honeycomb (**hcb**) network built in the trigonal *P*3 space group. The shape of the 6-member ring is distorted from the perfect hexagonal shape due to the nonlinear connections formed by the MPPD units. This observation paralleled our previously reported DFP-based COFs.^61−63^ The simulated PXRD pattern that best matched our experimental pattern corresponded to the structure with the stacking sequence ABC, resulting in cell parameters *a* = *b* = 33.0 Å and *c* = 10.4 Å. Accordingly, the intense diffraction peak at 2θ = 6.1° is attributed to the (110) plane (*d* = 16.50 Å). The diffraction signal centered at 25.8° assigned to the (003) plane is consistent with the formation of a π–π stacked layered structure,^64^ with an interlayer spacing of 0.35 nm, estimated from the structural mode (Figure. 1c).

**Figure 2:**
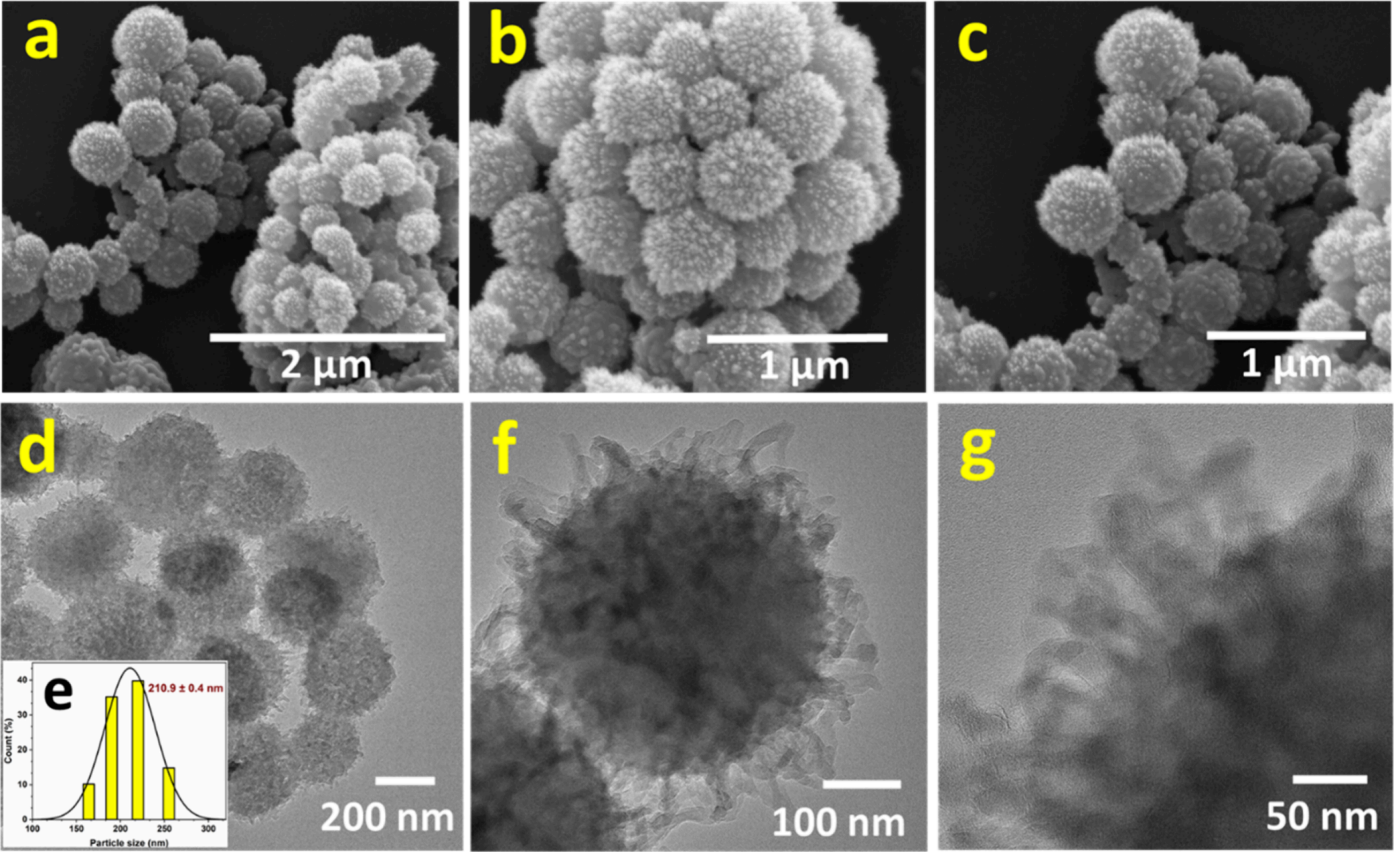
(a–c) Scanning electron microscopy images of TTASDFP at different scales. (d–g) High-resolution transmission electron microscopy images showing the sea-urchin morphology, with an average diameter of ∼210 ± 0.4 nm obtained via a Zetasizer Nano light scattering device.

The morphological properties of TTASDFP were analyzed by scanning electron microscopy (SEM) and high-resolution transmission electron microscopy (HR-TEM). SEM images showed that the COF displays a unique morphology that resembles a sea urchin (Figure. 2a–c). This morphology was also observed under HR-TEM, which showed that the particles are solid and have a uniform size, with an average diameter of ∼210 ± 0.4 nm (Figure. 2d–g).

The porosity and surface area of TTASDFP were also studied by measuring the adsorption and desorption isotherms of nitrogen gas at 77 K (Figure S4) From the type-II adsorption isotherms, the Brunauer–Emmett–Teller (BET) surface area of TTASDFP was relatively small (50.8 m^2^·g^–1^). The pore size distribution was determined using the density functional theory model (DFT) for the measured adsorption isotherm, which showed that TTASDFP has narrow pores with an average width of 1.3 nm, which is consistent with the pore size of 1.6 nm predicted from the proposed crystal structure (Figure S5). Moreover, the high thermal stability of the COF was confirmed by thermogravimetric analysis (TGA, Figure S6). The weight-loss curve displayed a minimal weight loss below 400 °C, while the greatest drop in the weight was observed in the range of 450–600 °C. This indicates the degradation of the framework network and crystallinity loss occurred first, followed by the material’s chemical degradation into volatile byproducts.^65,66^

## 4 Gold Capture Studies

### 4.1 Gold Removal Performance

**Figure 3:**
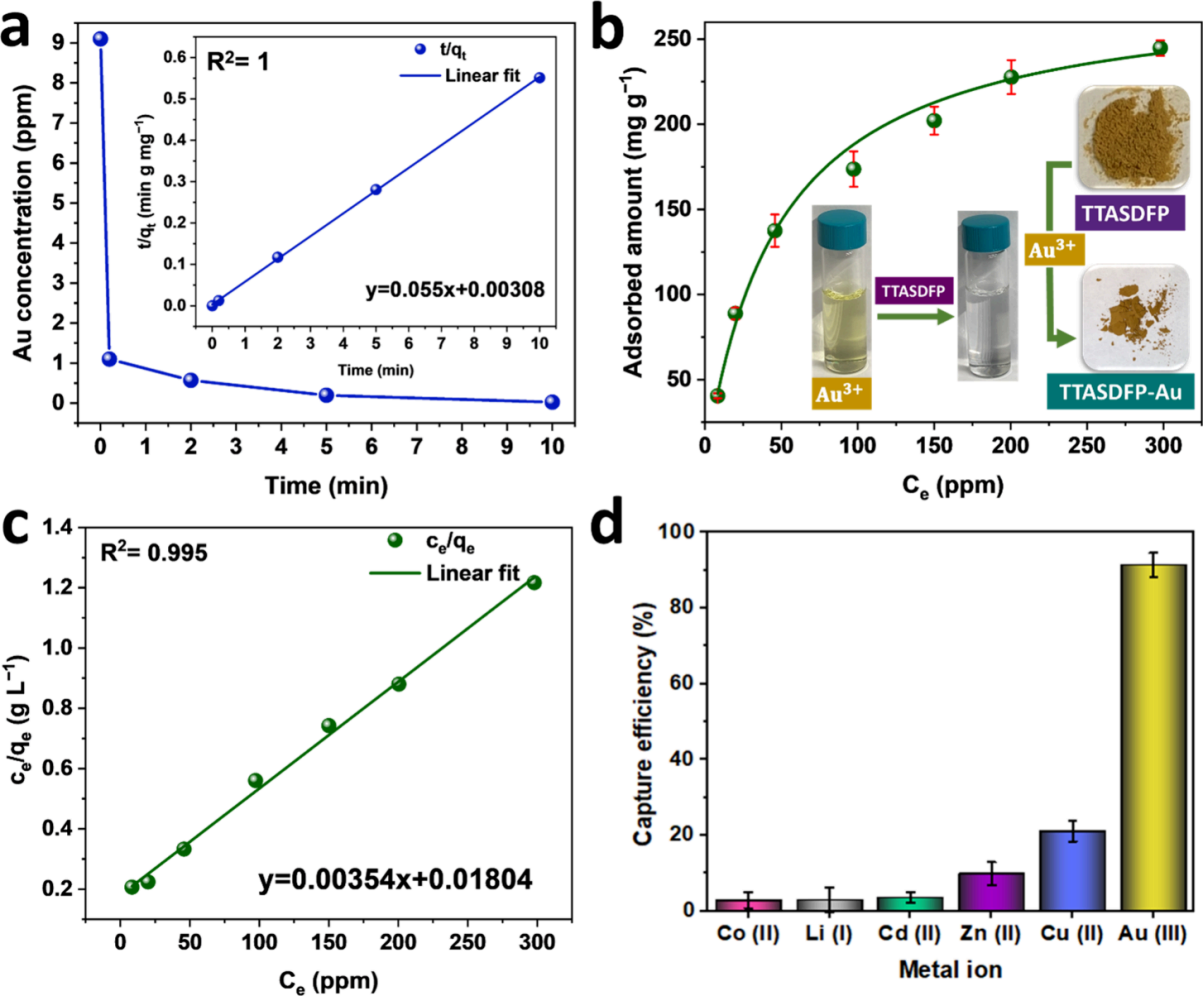
Gold removal performance. (a) Time-dependent gold adsorption via 500 mg L^−1^dose of TTASDFP with a starting concentration of 9 ppm, along with the pseudo-second-order kinetics model fitting. (b) Adsorption isotherm of gold ions via 200 mg L^−1^dose of the COF at initial concentrations of 9–300 ppm measured in triplicates. (c) Langmuir isotherm model fitting. (d) Au^3+^ capture efficiency in the presence of other metal ions (10 ppm each) after exposure for 90 min, which was measured in triplicates.

TTASDFP performance in trace Au^3+^ removal from water was evaluated at room temperature. Gold removal kinetics were first studied by subjecting the COF to a 9 ppm aqueous Au^3+^ solution prepared from gold(III) chloride hydrate. Results showed more than 98.7% of the gold ions were removed from water within 30 s, lowering the ion concentration to parts per billion levels (Figure. 3a). The kinetics for the Au^3+^ removal via our COF was faster compared to several reported COFs and adsorbents that required several minutes or hours to reach adsorption equilibrium, even when the gold concentration is approximate to 9 ppm (Table S2).^53^ The great performance is likely promoted by the strong gold–sulfur chelating, the porous COF's structural design, and the enhanced diffusion of the metal ions to the adsorbent's binding sites. The strong affinity of sulfur moieties to gold is primarily due to the higher surface polarity and negative charge of sulfur compared to atoms, such as carbon, in addition to the strong coordination of the sulfur’s lone pair electrons with the empty d orbits of the heavy metal ions.^67^ As for the structural design of TTASDFP, the extended network with permanent pores rich in anchored thioanisoles allowed gold ions to access the active adsorption sites more easily. This accessibility was further enhanced by the hydration of TTASDFP via a suspension in water prior to gold adsorption experiments. This adsorption methodology contributed to the faster ion diffusion and, hence, a fast Au^3+^ capture kinetics.^68,69^ Moreover, the AB stacking of the COF layers create confined spaces that might have contributed to trapping the metal ion within the adsorbent.^70^ To further understand the adsorption mechanism, the kinetics data were fitted to the pseudo-first- and -second-order models (Table S3). The correlation coefficient for the pseudo-second-order model (*R*^2^ = 1) was higher compared to the pseudo-first-order model (*R*^2^ = 0.690, Figure. S7), which implied that the gold capture followed pseudo-second-order kinetics. This indicated that the gold adsorption process on TTASDFP was chemisorption, which is most likely a result to the Au–S chelation.^53,71^

Additionally, the gold uptake at different concentrations was evaluated. The adsorption isotherm (Figure 3b) displayed an increased adsorption amount with an increase in the Au^3+^ concentration, with the maximum uptake reaching approximately 245 mg·g^–1^ at a relatively high concentration of 300 ppm. The Freundlich (Figure S8) and Langmuir isotherm models were used to analyze the isotherm data, and both of their calculated parameters are shown in Table S4. The results showed that the adsorption behavior fits well with the Langmuir model with a high correlation coefficient of 0.995 (Figure. 3c). This indicates the metal ions are adsorbed as monolayers on the surface of TTASDFP.^72,73^ To further understand the gold capture process during the adsorption isotherm, zeta potential was measured to monitor the surface charge of the COF with respect to the increasing Au^3+^ concentrations (Figure S9). There was an overall decrease in the ζ potential of TTASDFP to − 37.9 mV, which can be attributed to the uptake of Au^3+^ as the negatively charged [AuCl_4_]^–^ ions.^43^ This was supported by the elemental mapping conducted on the adsorbent at different gold concentrations, which displayed an increase in both gold and chloride contents (Figure S10, Table S5). In addition, the initial decline in the ζ potential followed by a temporary plateau line at gold concentrations of 9–50 ppm indicate that metal ions adopt adsorption on the COF’s outer surface first, followed by the pores of the adsorbent, with the maximum adsorption capacity reached around the concentration 300 ppm Au^3+^.^74^ Due to the presence of various metal ions in bodies of water, it is crucial for an adsorbent to be selective toward Au^3+^. Therefore, we studied the adsorption selectivity by exposing TTASDFP to an aqueous solution containing mixed metal ions consisting of Au^3+^, Co^2+^, Cd^2+^, Li^+^, Cu^2+^, and Zn^2+^, all at an initial concentration of 10 ppm (Figure. 3d). The COF proved to possess great selectivity toward gold ions, as 91% of Au^3+^ was captured, which was much higher when compared to the other competitive ions. The high selectivity of the COF for Au^3+^ is the result of the S–Au binding that is stronger than the other S–metal, as reported through previous studies.^50,51,75^

### 4.2 Post-Gold-Removal Analysis

**Figure 4:**
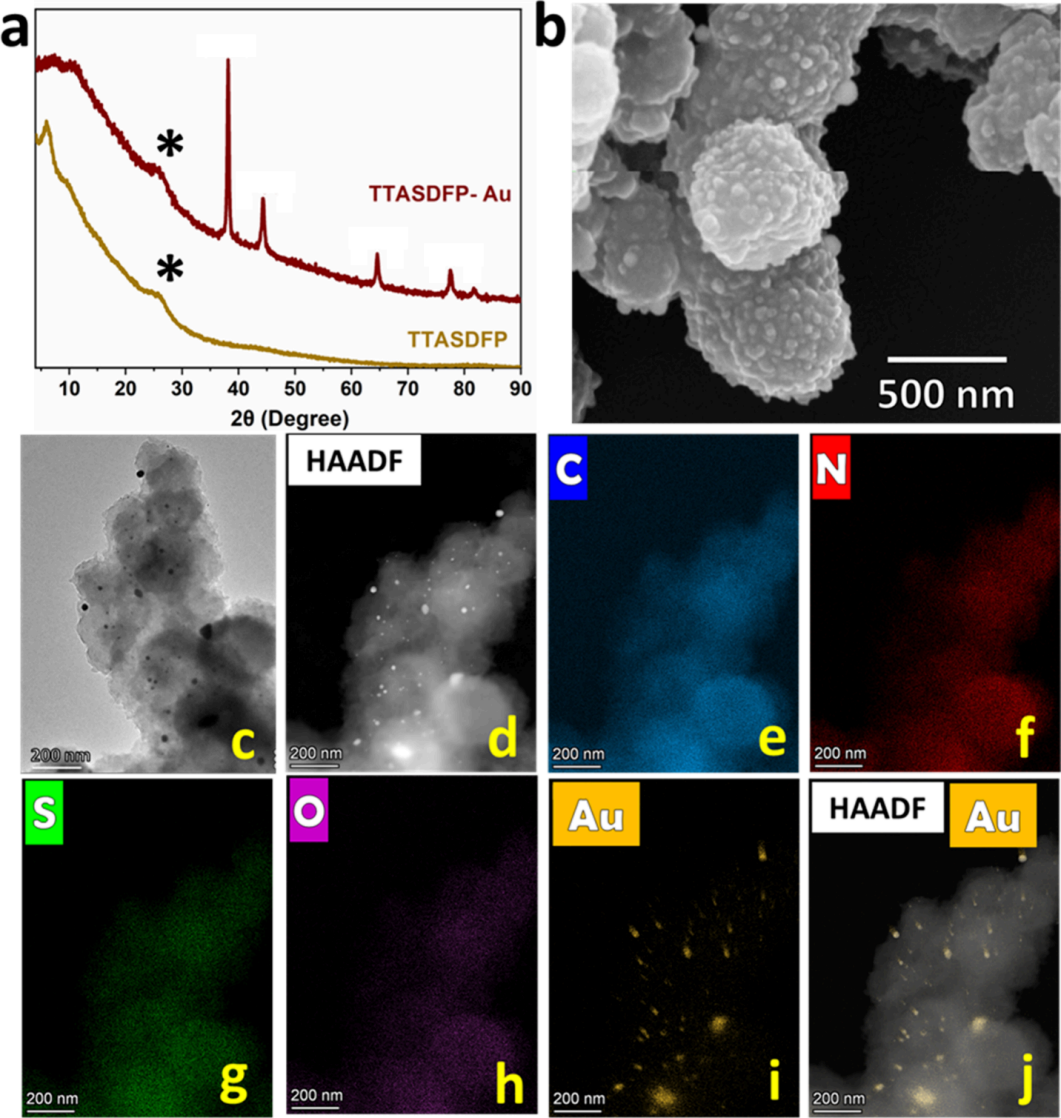
Post-gold-capture characterization. (a) Stacked experimental powder X-ray diffraction patterns of TTASDFP (orange) and TTASDFP-Au (red), showing the elemental diffraction peaks of Au. (b) SEM image of TTASDFP-Au. (c–j) SEM and HR-TEM images of TTASDFP-Au showing the adsorbed gold. (d–j) Elemental mapping of TTASDFP-Au.

To confirm the gold ion adsorption on the COF and verify the interaction between the analyte and TTASDFP, we analyzed the material obtained following Au^3+^ uptake (TTASDFP-Au). The PXRD pattern displayed a large reduction in the first peak intensity (6.1°), which can be attributed to the structural disturbance due to the adsorbed gold. However, the diffraction pattern maintained the peak at ∼26° corresponding to the π–π stacking, which indicates that the COF’s structure was mostly preserved and the π–π distance were largely intact.^42^ In addition, the presence of additional peaks at 38.0, 44.3, 64.4, and 77.5°, corresponding to Bragg reflections of (111), (200), (220), and (311), respectively, which are characteristic of gold nanocrystals (Figure 4a).^76^ The detection of gold crystals in the material is most likely due to the ability of Au^3+^ to self-reduce.^77,78^ SEM analysis revealed that TTASDFP-Au maintained its sea-urchin-like morphology (Figure 4b). Moreover, gold clusters of different sizes were observed by using HRTEM (Figure 4c). Energy-dispersive X-ray spectroscopy (EDS) elemental mapping of TTASDFP before and after adsorption also revealed the presence of gold in the material postexposure to Au^3+^ (Figures. 4d–j and S11).

**Figure 5:**
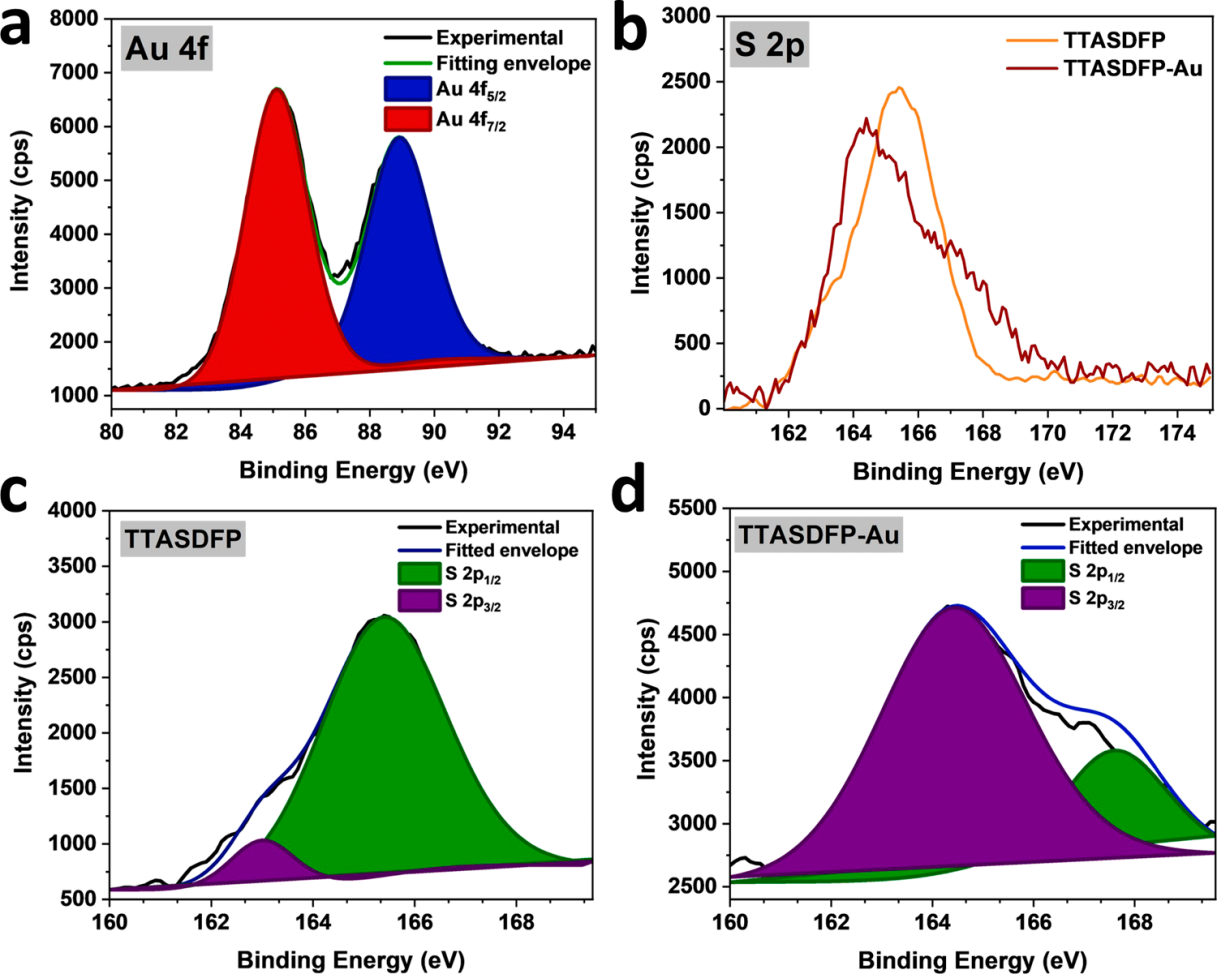
X-ray photoelectron spectroscopy (XPS) analyses: (a) High-resolution Au 4f spectrum for TTASDFP-Au with the deconvoluted results. (b) S 2p spectra of TTASDFP (red) and TTASDFP-Au (orange). (c, d) Deconvolutes of the S 2p signal collected for TTASDFP and TTASDF-Au.

The material was also characterized by X-ray photoelectron spectroscopy (XPS) to further analyze the interactions between the COF and Au^3+^. The XPS spectra of TTASDFP-Au contained the Au 4f peak that confirms successful gold uptake by the material (Figure S12). The Au 4f spectrum was further analyzed, and signal deconvolution displayed two-peak components at 85.1 and 88.8 eV, which correspond to Au^3+^ 4f_7/2_ and Au^3+^ 4f_5/2_, respectively (Figure 5a).^79^ In addition, the gold interaction with the sulfur moieties was confirmed by the decrease of the overall S 2p signal by ∼1 eV pos- gold-adsorption, which is parallel to what was observed for other reported S–Au^3+^ systems (Figure 5b).^53^ Moreover, curve-fitting analysis of the S 2p signal showed that the spectra had two deconvoluted peaks assigned as S 2p_3/2_ and S 2p_1/2_, which were attributed to sulfur atoms bound and unbounded gold atoms, respectively (Figure. 5c,d).^79,80^ A comparison of the spectra showed that the S 2p_3/2_ peak experienced an increase in intensity, which supports the conclusion that the gold adsorption mechanism is driven by chemisorption, as chelate complexes occur between the thioanisole’s sulfur atoms and gold ions.^81^

### 4.3 Post-Gold-Removal Regeneration

**Figure 6:**
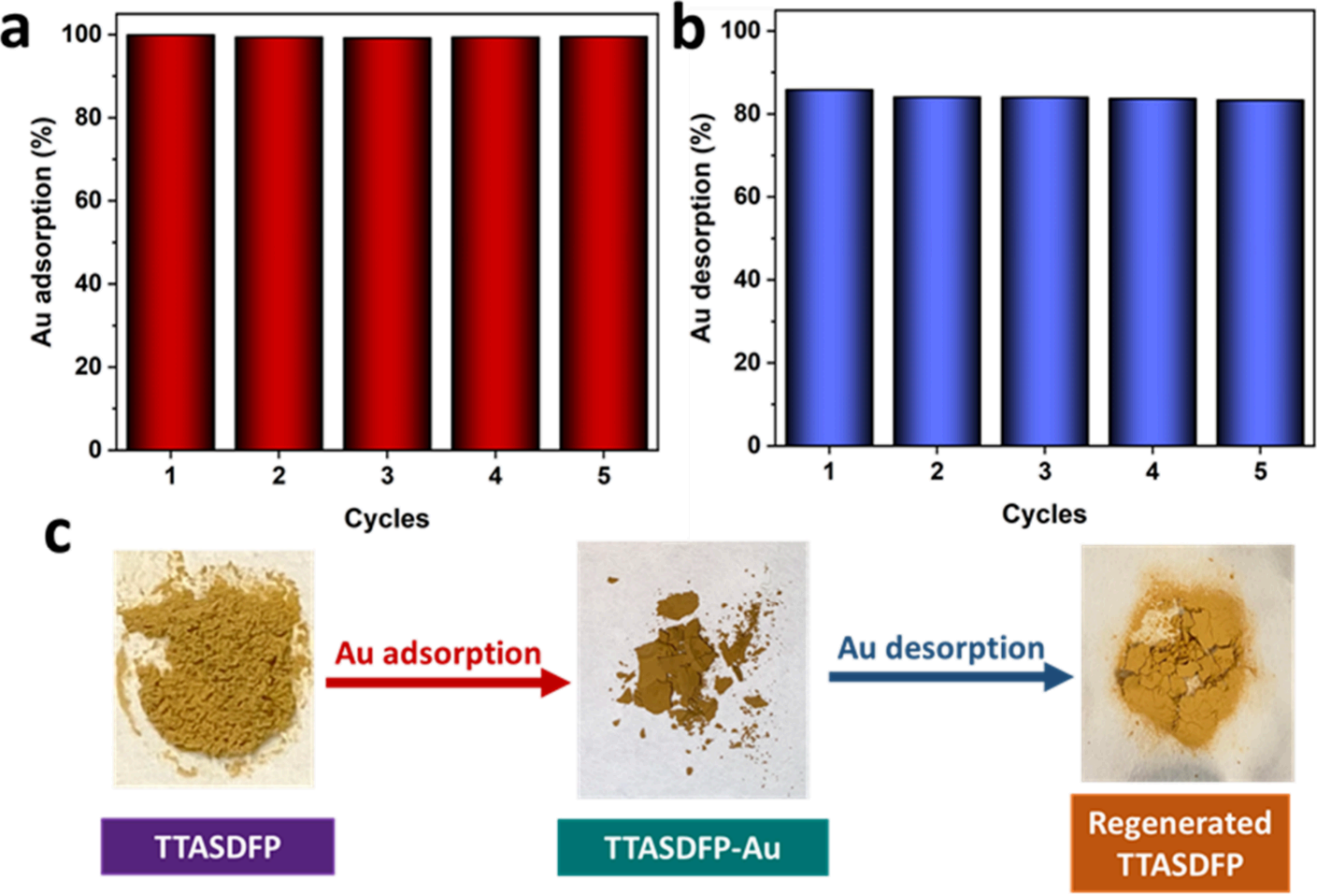
(a) Adsorption and (b) desorption cycles of gold via TTASDFP. (c) Images of TTASDFP powder during the adsorption and desorption cycles.

On account of the long periodic reusability is an essential property for an adsorbent; the recyclability of TTASDFP was also assessed. The COF performed excellently following regeneration, as more than 99% of a 100 ppm Au^3+^ solution was successfully removed in 5 consecutive cycles (Figure 6a). Between adsorption cycles, the captured gold was effectively desorbed using a 0.1 M thiourea solution in 0.2 M HCl, as acidic thiourea solutions are known for their complexation with gold and ability to trigger gold leaching.^82^ Moreover, TTASDFP proved to be stable following treatment with the solution, making it a suitable method for regeneration, as both the PXRD pattern and the morphology remained largely retained (Figure S13). The results showed that more than 82% of the adsorbed gold was successfully extracted from the COF during the 5 regeneration cycles (Figure 6b). The 17% difference in the desorption compared to the adsorption can be attributed to the remaining gold in the material after regeneration, a consequence of strong S–Au binding. In addition, the overall slight decrease in the adsorption and desorption percentages might be due to a loss in COF mass throughout the cycles and due to some binding sites being already occupied with gold. Nevertheless, the material showcased great durability, especially with the structure of TTASDFP appearing preserved post-regeneration, as the ^13^C NMR spectrum of the material was reproduced (Figure S14). Interestingly, the recycled COF also sustained its characteristic sea-urchin morphology, as seen from its SEM images (Figure S15a), showing the effectiveness of the regeneration process. TTASDFP also recovered most of its PXRD pattern after regeneration, indicating the release of trapped gold ions in the matrix. However, the pattern displays some gold traces that could have been occupying some of the COF’s binding sites (Figure S15b). Nevertheless, the material remains regeneratable and promising as a reusable sorbent for gold ions.

### 4.4 Extraction of ppb Levels of Au

**Figure 7:**
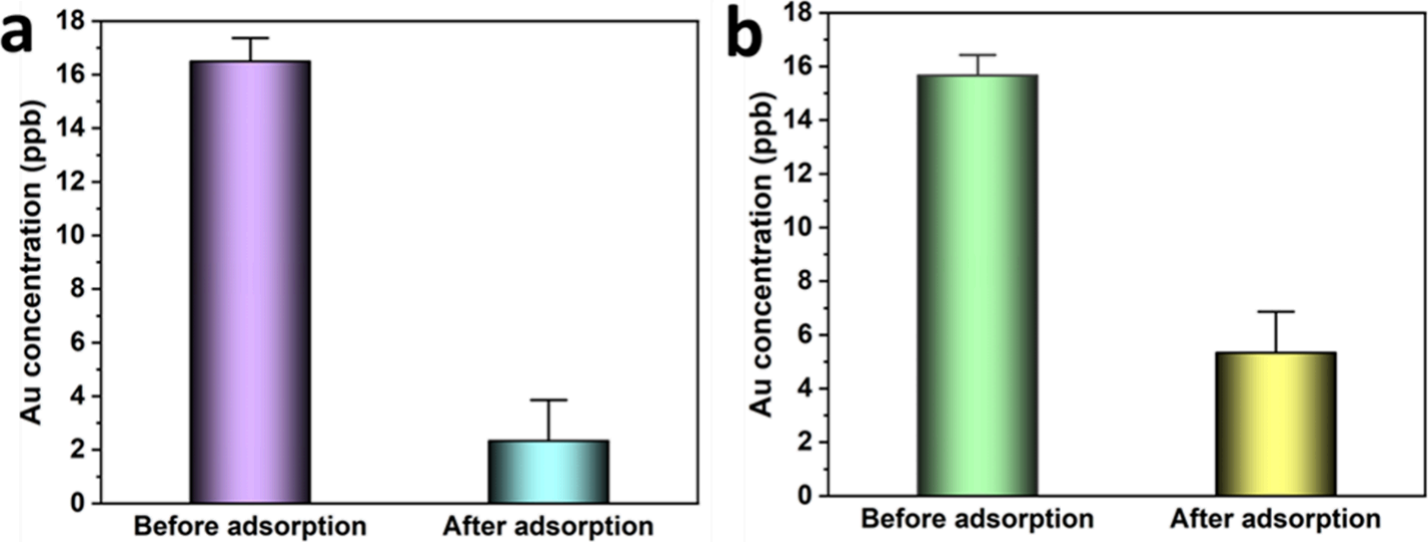
Concentration of Au^3+^ before and after treatment with TTASDFP in (a) a sodium-free environment and (b) in the presence of >10800 ppm NaCl.

To evaluate the potential for our COF to capture gold ions from wastewater, we investigated the ability of the material to remove parts per billion amounts of Au^3+^. First, the removal performance was tested in a control experiment where only gold ions with an initial concentration of 16 ppb were present in the system. The results show that TTASDFP was able to remove about 88% of the metal ions (Figure 7a). The successful capture of gold traces highlights the importance of the closely arranged thioanisole groups within the COFs pores, which bind strongly to gold ions. The results also display the contribution of the COF’s hydration via suspension in water toward achieving ppb level adsorption. Adsorption of gold in the ppb range was also tested in a saline environment containing NaCl (10800 ppm of Na^+^), a typical concentration found in seawater.^83^ Even in the presence of high sodium concentrations, the COF was able to adsorb 69% of the gold ions from a solution with an initial concentration of 16 ppb (Figure 7b). Since seawater contains other soft metals, such as copper, at much higher concentrations,^84^ we investigated TTASDFP performance in capturing gold from a 16 ppb concentration in the same saline conditions, and in the presence of 806 ppb Cu^2+^, which is approximately 8 times the excess levels found in copper-contaminated coastal waters.^85^ Despite the presence of excess copper ions, TTASDFP successfully replicated the ppb level capture performance, as about 70% of gold ions that were removed, compared to only 13.5% of copper ions being captured (Figure S16). These results showcase that the COF is still able to capture ultralow trace amounts of gold, even in the presence of large quantities of interfering ions.

## 5 Conclusions

In summary, we have presented the preparation and characterization of a porous imine-based COF, TTASDFP, which has an excellent ability to capture gold ions from water. The synthesis of thioanisole-rich COF by microwave irradiation was relatively easy and provided a high yield. TTASDFP effectively and rapidly adsorbed gold ions in the ppm range, with more than 98% uptake from a 9 ppm Au^3+^ solution within 30 s. The removal kinetics proved to be faster compared to several other known COFs and adsorbents. The COF also selectively removed gold ions from mixtures containing additional ions in the solution matrix such as Cd^2+^, Co^2+^, Cu^2+^, Li^+^, and Zn^2+^. In addition, the adsorption performance of TTASDFP was maintained following 5 cycles of regeneration, a result which augurs well for the material’s sustainability. Recyclability of the adsorbent was also highlighted by the maintained morphology of TTASDFP during the gold capture and regeneration processes. Our material was also capable of capturing Au^3+^ in the range of 1–16 ppb, even in media containing high levels of NaCl and Cu^2+^. These results highlight the impact that the material’s porosity and functionalized pore surface have on its gold sorption efficiency. Such a COF can potentially be used as a durable adsorbent to collect ultralow traces of gold present in wastewater, an untapped resource with the potential to positively impact the environment and the economy.

## Supporting Information Available

Materials, COF synthetic procedure, gold adsorption methodology and experiments, FT-IR spectra, electron microscopy images, COF ^13^C CP/MAS NMR spectrum, TGA, surface area and porosity analyses, XPS spectra, COF recyclability, and the ^13^C CP/MAS NMR, SEM, and PXRD assessments of the COF’s stability postregeneration (PDF)
Crystal structure data of the COF (CIF)

## Supplementary Material

am4c03685_si_001.pdf

am4c03685_si_002.cif
