# Supplementary material for: Enhanced Removal of Ultratrace Levels of Gold from Wastewater Using Sulfur-Rich Covalent Organic Frameworks
Source: ACS Appl Mater Interfaces. 2024 Jun 1;17(12):17794–803. doi: 10.1021/acsami.4c03685 (PMC11955949; doi:10.1021/acsami.4c03685)
Supplement: Supplementary file 1 — am4c03685_si_001.pdf [file am4c03685_si_001.pdf]

Supporting Information

Enhanced Removal of Ultra-Trace Levels of Gold from Wastewater Using Sulfur-Rich Covalent Organic Frameworks

Salma Abubakar,[1] Gobinda Das,[1] Thirumurugan Prakasam,[1] Asmaa Jrad,[1,2] Felipe Gándara,[3] Sabu Varghese,[4] Thomas Delclos,[5] Mark A. Olson,[6] and Ali Trabolsi [1,2*]

[1] Science Division, New York University Abu Dhabi, Saadiyat Island, 129188, Abu Dhabi, United Arab Emirates

[2] Water Research Centre, New York University Abu Dhabi, Saadiyat Island, 129118, Abu Dhabi, United Arab Emirates

[3] Materials Science Institute of Madrid − CSIC, Sor Juana Inés de la Cruz 3, 28049 Madrid, Spain

[4] CTP, New York University Abu Dhabi, Saadiyat Island, 129188 Abu Dhabi, United Arab Emirates

[5] Materials and Surface Core Labs, Khalifa University of Science and Technology, 127788, Abu Dhabi, United Arab Emirates

[6] Department of Physical and Environmental Sciences, Texas A&M University Corpus Christi, 6300 Ocean Dr., Corpus Christi, TX 78412 USA

Email address*: ali.trabolsi@nyu.edu

1. Materials

2. Instruments and Methods

3. Synthesis of TTASDFP COF

4. Gold Adsorption Experiments and Methods

Figure. S1: Structure of TTASDFP's pores

Figure. S2: FT-IR Spectra of TTASDFP and its precursors

Figure. S3: Cross-Polarization Magic Angle Spinning ^{13}C Nuclear Magnetic Resonance (^{13}C CP/MAS NMR) spectrum of TTASDFP

Figure. S4: N_2 Adsorption isotherm of TTASDFP

Figure. S5: Pore size distribution of TTASDFP

Figure. S6: Thermogravimetric Analysis (TGA) of TTASDFP

Figure. S7: The pseudo-first order kinetic model fitting for Au^{3+} removal

Figure. S8: Freundlich isothermal model fitting for Au^{3+} removal

Figure. S9: Zeta potential of TTASDFP subjected to different Au^{3+} concentrations

Figure. S10: Elemental mapping of TTASDFP with different Au^{3+} adsorption concentrations.

Figure. S11: Energy-Dispersive X-ray Spectroscopy (EDS) analysis of TTASDFP

Figure. S12: X-ray Photoelectron Spectroscopy (XPS) Survey of TTASDFP and TTASDFP-Au

Figure. S13: TTASDFP stability under regeneration conditions: PXRD and TEM

Figure. S14: ^{13}C CP/MAS NMR analysis of TTASDFP and regenerated TTASDFP

Figure. S15: SEM and PXRD analyses of the regenerated TTASDFP

Figure. S16: Removal of ppb levels of Au^{3+} in the presence of excess NaCl and Cu^{2+}

Table. S1: Au^{3+} concentrations targeted by previously reported sulfur-based COFs during adsorption studies

Table. S2: Comparison of Au^{3+} removal performance for reported adsorbents

Table. S3: Au^{3+} adsorption kinetic models for TTASDFP

Table. S4: Au^{3+} adsorption isothermal-models for TTASDFP

Table. S5: Atomic fractions obtained via STEM at different Au^{3+} concentrations

5. Crystal data of TTASDFP COF model in a cif compatible format

1. Materials

All reagents and compounds used for the synthesis of the covalent organic framework and the preparation of metal ion solutions targeted for adsorption studies ($HAuCl_4$. xH_2O, Zn $(NO_3)_2 \cdot 6H_2O$, Cu $(NO_3)_2$, $LiNO_3$, Co $(NO_3)_2 \cdot 6H_2O$, Cd $(NO_3)_2 \cdot 4H_2O$, NaCl) were all purchased from Sigma-Aldrich, Fisher Scientific, and Laboratory Scientific Supplies and utilized without further purification. The synthesis of 2,4,6-tris(4-aminophenyl)-1,3,5-triazine (TAP), which is the amine-based linker used for preparing TTASDP COF, was conducted following a previously reported procedure.[1] 4-(4-(methylthio) phenyl) pyridine-2,6-dicarbaldehyde (MPPD) was also prepared following a procedure we previously reported.[2]

2. Instruments and Methods

2.1 Routine liquid proton Nuclear Magnetic Resonance (^{1}H NMR) spectra were recorded on a Bruker Avance spectrometer at 25 °C and a frequency of 500 megahertz (MHz). The chemical shifts (ppm) are reported relative to the signals corresponding to the residual solvents ($CHCl_3$: δ = 7.24 ppm, DMSO: δ = 2.5 ppm). The multiplicity of the ^{1}H NMR peaks are abbreviated as the following: s (singlet) and d (doublet).

2.2 Fourier Transform Infrared (FT-IR) spectroscopic studies were carried out on a Agilent FT-IR 670/630 spectrometer.

2.3 Powder X ray Diffraction (PXRD) patterns were recorded on a Malvern Panalytical Empyrean 2 diffractometer using Cu Kα radiation (scan step size: 0.053°, time per step: 99.45 s). The crystal model was built with Biovia Materials Studio software package, and the corresponding PXRD pattern was calculated with the Reflex module of the program.

2.4 Thermogravimetric Analysis (TGA) was performed on a Q600 over the temperature range of 25- 900 ºC under an Argon atmosphere with a heating rate of 10 $°C\cdot min^{-1}$.

2.5 Magic Angle Spinning (MAS) solid-state NMR experiments were carried out on a Bruker Avance-HD 600 MHz spectrometer (Bruker BioSpin GmbH) operating at a static field of 14.1 T using a 4.0 mm MAS probe. Samples were packed into 4.0 mm zirconia rotors and were spun at a MAS frequency of 14 kHz.

2.6 ^{1}H-^{13}C Cross-Polarization Magic Angle Spinning (CP/MAS) experiments were performed using a standard linearly ramped cross-polarization pulse sequence. ^{13}C chemical shifts were externally referenced to the adamantane CH_2 signal at 38.48 ppm. NMR data were processed using TopSpin software (Bruker BioSpin GmbH).

2.7 Scanning Electron Microscopy (SEM) images were obtained from a ThermoFischer Quanta3D Scanning Electron Microscope and the samples were prepared by dropping a solution of dispersed COF in ethanol onto a silicon plate. The samples were allowed to dry prior to imaging.

2.8 High Resolution Transmission Electron Microscopy (HR-TEM) images and diffraction patterns were obtained using a ThermoFischer TAlos F200X. The samples were prepared by dropping a solution of dispersed COF in ethanol onto carbon grids coated on 400 mesh copper. The samples were allowed to dry prior to imaging.

2.9 The Brunauer-Emmett-Teller (BET) method was used to analyze surface properties. N_2 adsorption/desorption isotherms was recorded at 77 K up to 1 bar via a manometric Micromeritics 3Flex gas sorption analyzer. The sample was fully activated prior to measurement using a degas station by heating at 100 °C for 22 hours. The pore distribution was calculated using the Nonlocal Density Functional Theory (NLDFT).

2.10 Inductively Coupled Plasma Mass Spectrometry (ICP-MS) analysis was conducted on an Agilent 7800/ICP-MS.

2.11 Particle size distribution and zeta potential measurements were conducted on MalvernNano Zetasizer via dynamic light scattering (DLS) technique.

2.12 X-ray photoelectron spectroscopy (XPS) analysis was conducted on a ThermoFisher Escalab Xi+ using a monochromatic X-ray source (Al) and a charge compensation Flood Gun (Dual bean electron / ion). Survey scans were run from -10 eV to 1350 eV. Samples immobilized on carbon tape gave the best results.

3. Synthesis of TTASDFP COF

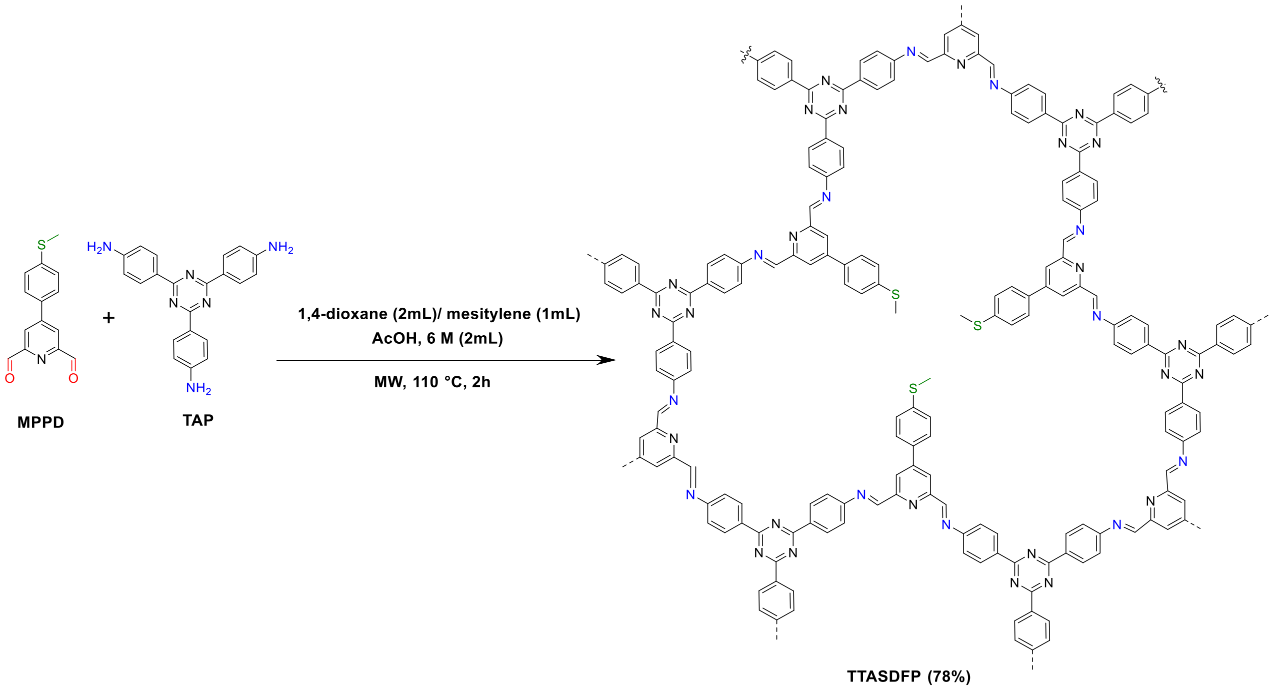

Scheme. S1: Synthetic scheme and chemical structure of TTASDFP COF which was obtained under microwave (MW) irradiation, with a synthetic yield of 78%.

The synthesis of TTASDFP was conducted under microwave irradiation. 4-(4-(methylthio) phenyl) pyridine-2,6-dicarbaldehyde (MPPD, 46.26 mg, 0.09 mmol) and 2,4,6-tris(4-aminophenyl)-1,3,5-triazine (TAP, 42.4 mg, 0.06 mmol) were placed in a 35 mL microwave reaction vessel. Then, anhydrous 1,4 dioxane (2 mL) and mesitylene (1 mL) were added and the mixture and sonicated until the solids were dissolved. A 2 mL aliquot was taken from an aqueous solution of 6 M acetic acid and added to the mixture, resulting in the immediate formation of a yellow precipitate. The mixture was then subjected to microwave irradiation under stirring at 110 °C for 2 hours. After cooling to room temperature, the precipitate was

isolated through centrifugation and washed with ethanol (20 mL, 3 times) before drying in a vacuum oven at 50 °C. The synthetic yield was approximately 78%.

4. Gold Adsorption Experiments and Methods

Aqueous solutions of metal ions with different concentrations were prepared by dissolving metal salts in suitable amounts of Milli-Q water to obtain stock solutions. Unless otherwise specified, the concentration of the metal ions in aqueous solutions was determined using ICP-MS. All adsorption experiments were conducted in aqueous solution, at room temperature and under stirring at 500 revolutions per minute (rpm). Prior to the experiments, the vacuum-dried TTASDFP was rehydrated by suspending 80 mg of the adsorbent in 80 mL Milli-Q water to yield a 1000 $mg \cdot L^{-1}$ suspension. This suspension was sonicated for 10 minutes and stirred for 3 hours at 500 rpm to maximize the analytes' accessibility to the COF's sorption sites.[3, 4] The suspension was then used as the adsorbent stock for the ion removal experiment, from which aliquots were taken to achieve the targeted adsorbent concentrations.

4.1 Au^{3+} adsorption kinetics: The batch experiments were conducted in a 24 mL mixture containing an adsorbent dose of 500 $mg \cdot L^{-1}$. The suspension was spiked with Au^{3+} to generate an initial concentration of 9 ppm. Then, 3 mL aliquots were collected at the predetermined times of 0, 0.5, 2, 5, 10, 20, 30, 45, and 60 minutes. These aliquots were filtered with a 0.22 µm membrane filter, and the filtrate was analyzed by ICP-MS. The experimental results were plotted and fitted to both a pseudo-first order (equation 2) and pseudo-second-order (equation 3) adsorption kinetic models, as shown below:

$$q_t = \frac{(C_i - C_t)\,V}{m} \qquad (1)$$

$$\log(q_e - q_t) = log q_e - \frac{k_1}{2.303} t \qquad (2)$$

$$\frac{t}{q_t} = \frac{1}{k_2 q_e^2} + \frac{1}{q_e} t \qquad (3)$$

In which q_t is the adsorption quantity ($mg \cdot g^{-1}$) at a particular time (t, min), C_i and C_t are the initial concentration and final concentrations of Au^{3+} at a given time, respectively (ppm). V is the solution volume (L), m is the adsorbent mass (g), q_e is the equilibrium adsorption capacity ($mg \cdot g^{-1}$), and k_2 ($g \cdot mg^{-1} \cdot min^{-1}$) is the rate constant of pseudo-second order adsorption kinetics.

4.2 Au^{3+} adsorption isotherm: The experiments were performed in 5 mL samples containing an adsorbent concentration of 200 $mg \cdot L^{-1}$. The Au^{3+} concentration range in the isotherm adsorption experiments was 9-300 ppm. The adsorption solutions were under stirring for 12 hours before samples were taken and filtered with 0.22 µm membrane filter. The filtrates were diluted prior to analyzing under ICP-MS. The adsorption capacity at equilibrium (qe, $mg \cdot g^{-1}$) was determined using the equation below (equation 4). The isotherm was then fitted with Langmuir (equation 5) and Freundlich (equation 6) models.

.

$$q_e = \frac{(C_i - C_e)V}{m} \quad (4)$$

$$\frac{C_e}{q_e} = \frac{1}{q_{max} \times K_L} + \frac{C_e}{q_{max}} \quad (5)$$

$$lnq_e = lnK_F + \frac{1}{n} lnC_e \quad (6)$$

4.3 Au^{3+} adsorption selectivity: A 20 mL solution was prepared containing equimolar amounts of mixed ions of Au^{3+}, Zn^{2+}, Cu^{2+}, Cd^{2+}, Li^{+}, and Co^{2+}, all at a starting concentration of 10 ppm. The adsorbent was introduced to the solution to generate a final dose of 200 mg·L^{-1}, and the mixture was stirred for 90 minutes. Then, the mixture was filtered with a 0.22 µm membrane filter before analyzing the filtrates using ICP-MS to determine the capture efficiency percentage for each metal ion, as seen in the equation below:

$$Capture\ efficency = \frac{(C_i - C)}{C_i} \times 100 \quad (4)$$

4.4 Regeneration: TTASDFP (40 mg) was added to a glass container containing 100 ppm Au^{3+} (30 mL). The mixture was stirred at room temperature for 2 hours, and then filtered through filter paper before washing with Milli-Q water (20 mL). The filtrate was analyzed with ICP-MS to determine the gold removal percentage. The desorption of gold ions from the material was done by stirring the recovered material in 20 mL solution of 0.1 M thiourea and 0.2 M HCl for 5 hours. The mixture was then filtered and washed with water (20 mL). Desorption was monitored by measuring the gold content in the regenerated COF using ICP-MS after the COF digestion in concentrated nitric acid. The dried recycled TTASDFP was used for the next cycle, and the process was repeated for 5 cycles.

4.5 ppb Au^{3+} adsorption studies: Two sets of experiments were conducted in 5 mL solutions, a control experiment, and an experiment in the presence of NaCl. In the control experiment, a 5 mL suspension containing an adsorbent dose of 500 mg·L^{-1} was spiked with Au^{3+} to reach a starting concentration of 16 ppb. The mixture was allowed to stir for 12 hours before filtering the mixture with a 0.22 µm membrane filter prior to analysis by ICP-MS. For the experiments conducted in the presence of NaCl, 5 mL solutions of NaCl were prepared to make 10800 ppm Na^{+}. The solutions were later spiked with Au^{3+} to reach a starting concentration of 16 ppb before introducing 500 mg·L^{-1} adsorbent dose. Similar to the control experiments, these solutions were left to stir for 12 hours before filtering the solutions with a 0.22 µm membrane filter and analyzing the filtrates using ICP-MS. For the experiments conducted in the presence of NaCl and Cu^{2+}, 5 mL solutions of NaCl were prepared to make 10800 ppm Na^{+} and 806 Cu^{2+}. The solutions were later spiked with Au^{3+} to reach a starting concentration of 16 ppb before introducing 500 mg·L^{-1} adsorbent dose. Similar to the control experiments, these solutions were left to stir for 12 hours before filtering them with a 0.22 µm membrane filter and analyzing the filtrates using ICP-MS.

Structure of TTASDFP's pores

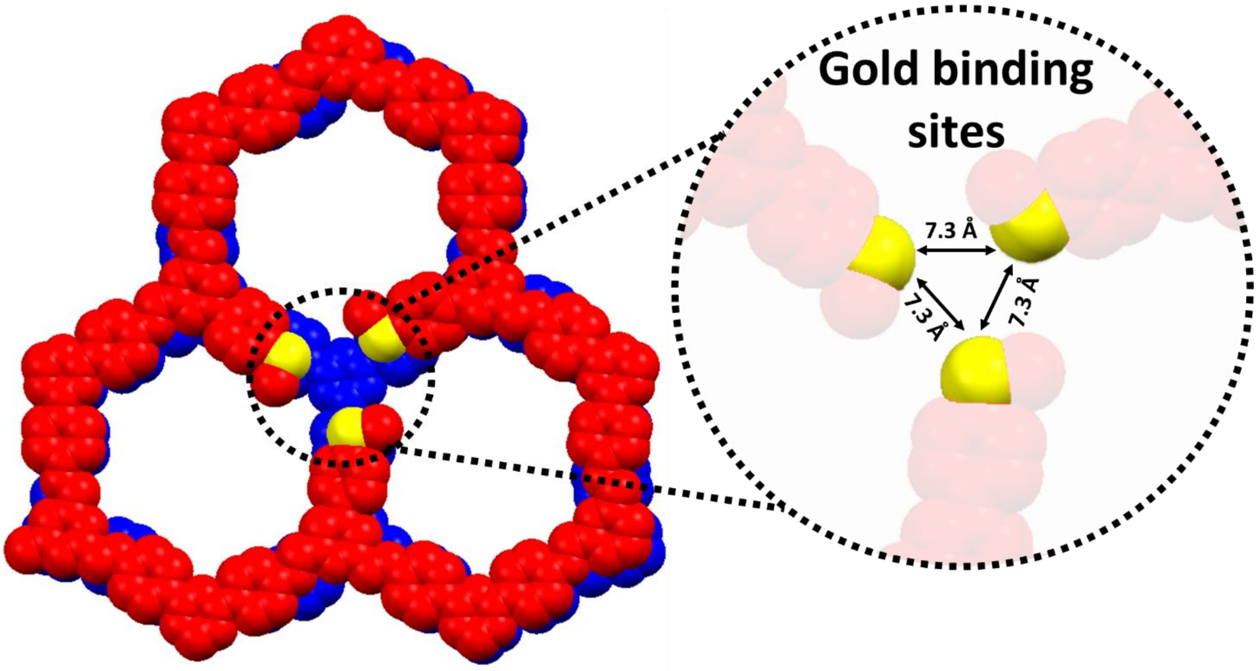

Figure. S1: Structure of TTASDFP COF's pores displaying the sulfur sites that serve as active sites for capturing gold ions. The different layers of the COF are differentiated by blue and red colors.

FT-IR Spectra of TTASDFP and its precursors

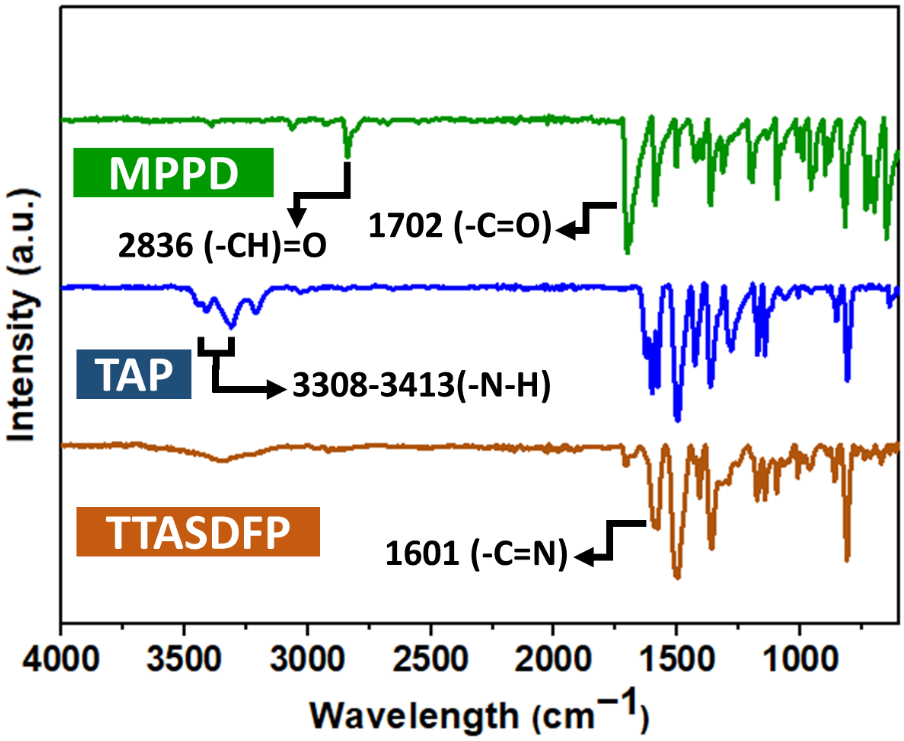

Figure. S2: Stacked FT-IR spectra of MPPD (green), TAP (blue), and TTASDFP COF (orange).

Cross-Polarization Magic Angle Spinning 13C Nuclear Magnetic Resonance (^{13}C CP/MAS NMR) spectrum of TTASDFP

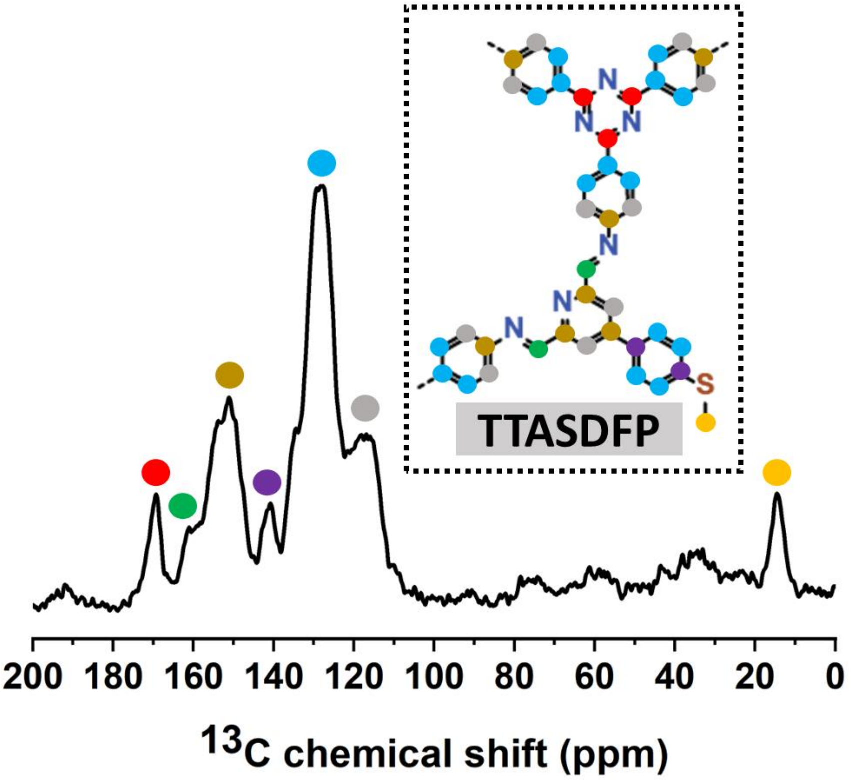

Figure. S3: ^{13}C CP/MAS NMR spectrum of TTASDFP.

N_2 Adsorption isotherm of TTASDFP

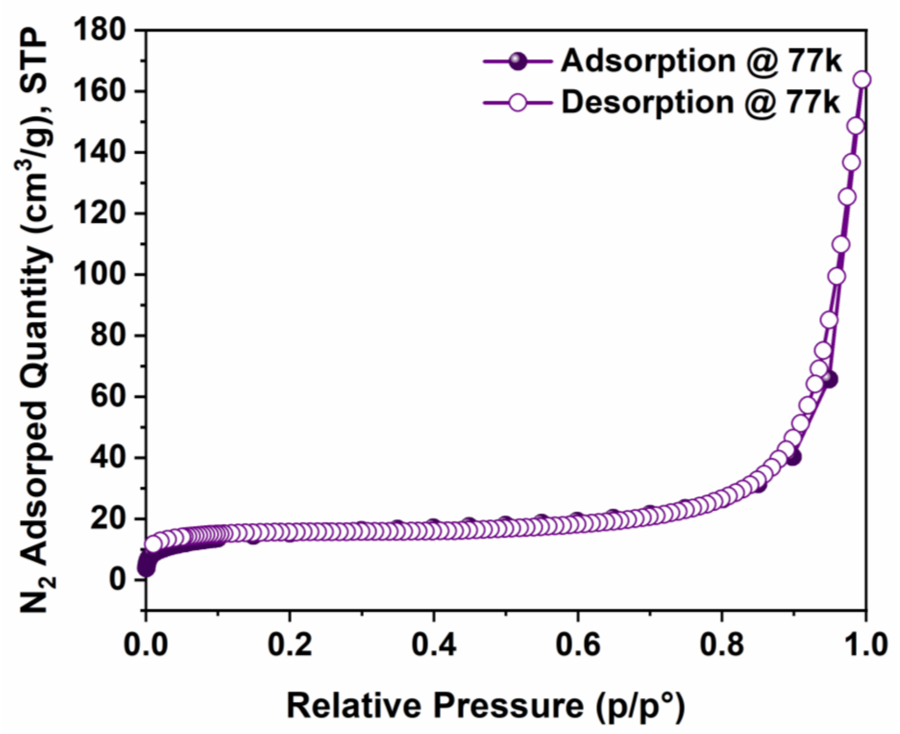

Figure. S4: N_2 adsorption isotherm of TTASDFP after degassing at 100 ºC for 1,300 min.

Pore size distribution of TTASDFP

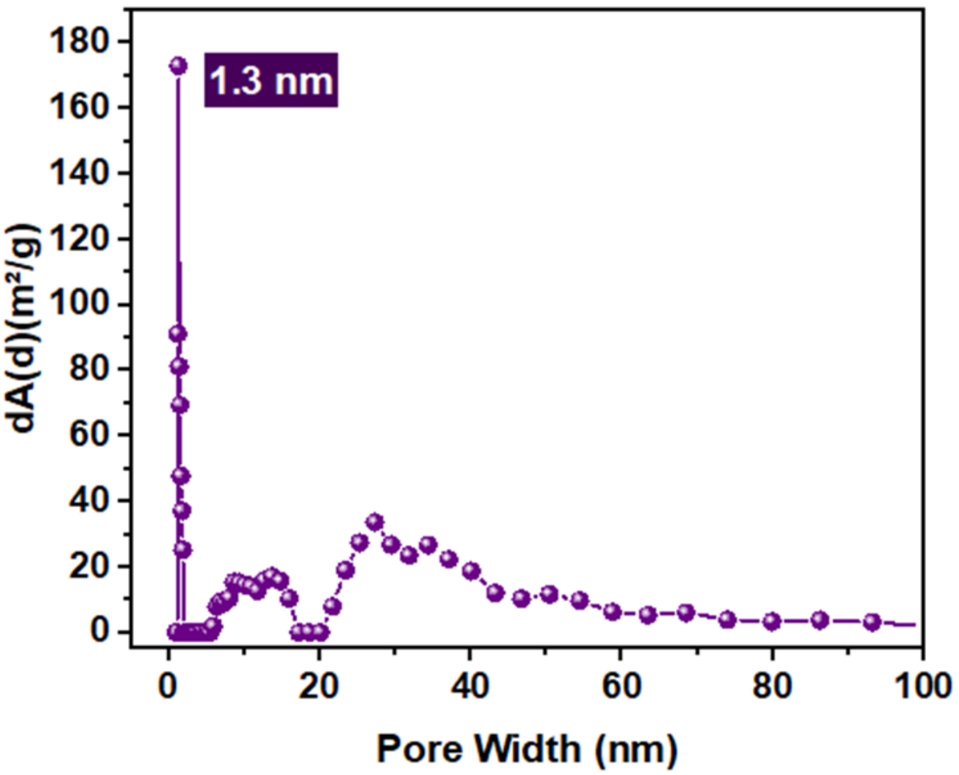

Figure. S5: Pore size distribution of TTASDFP.

Thermogravimetric Analysis (TGA) of TTASDFP

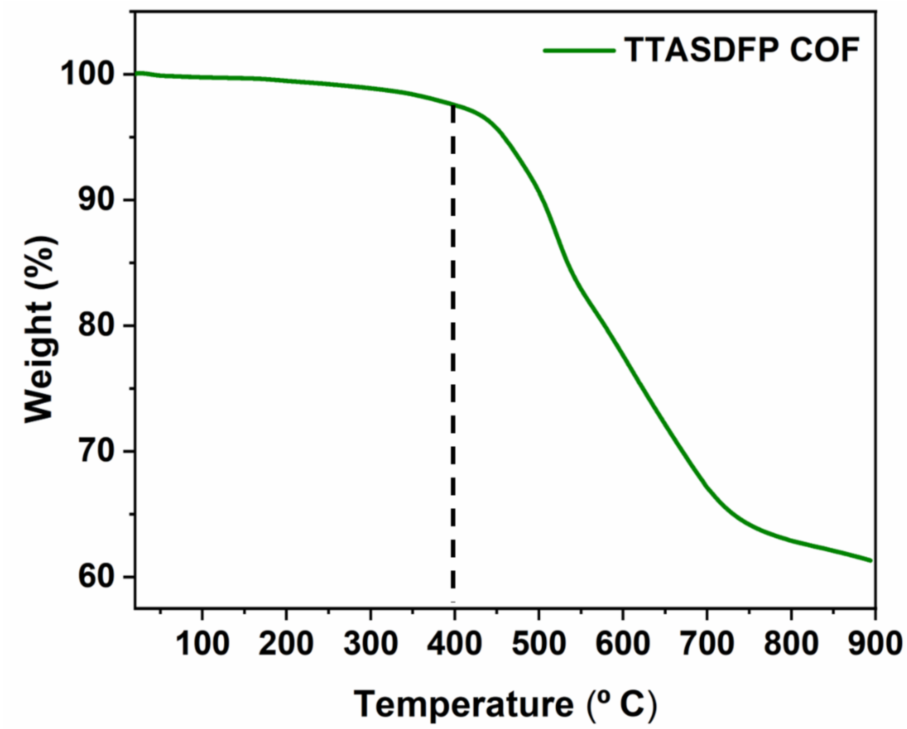

Figure. S6: TGA analysis of TTASDFP.

The pseudo-first order kinetic model fitting for Au3+ removal

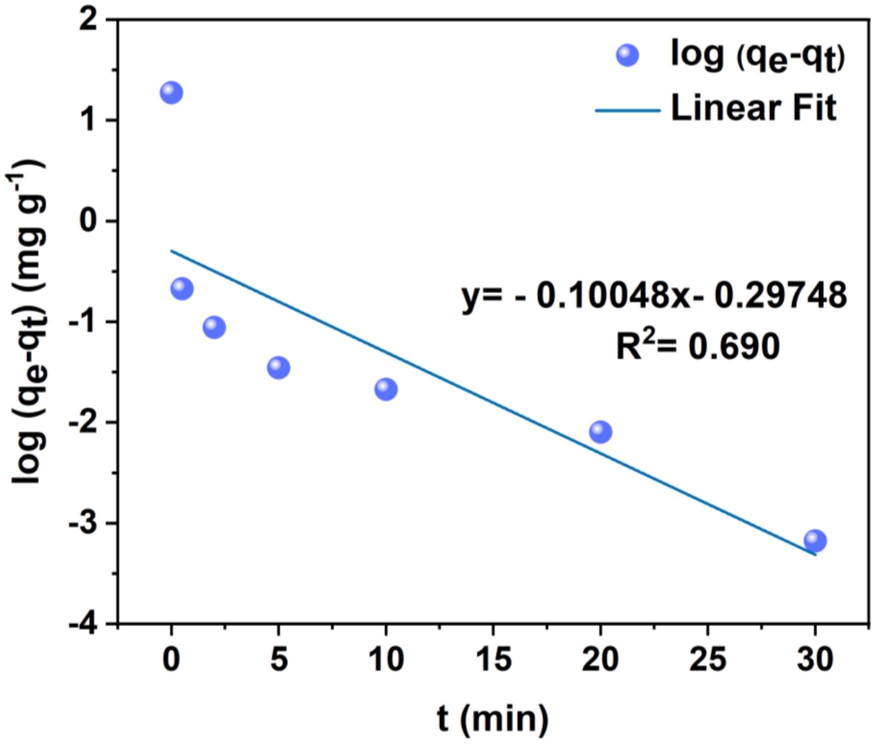

Figure. S7: The pseudo-first order kinetic model fitting for Au^{3+} removal.

Freundlich isothermal model fitting for Au^{3+} removal

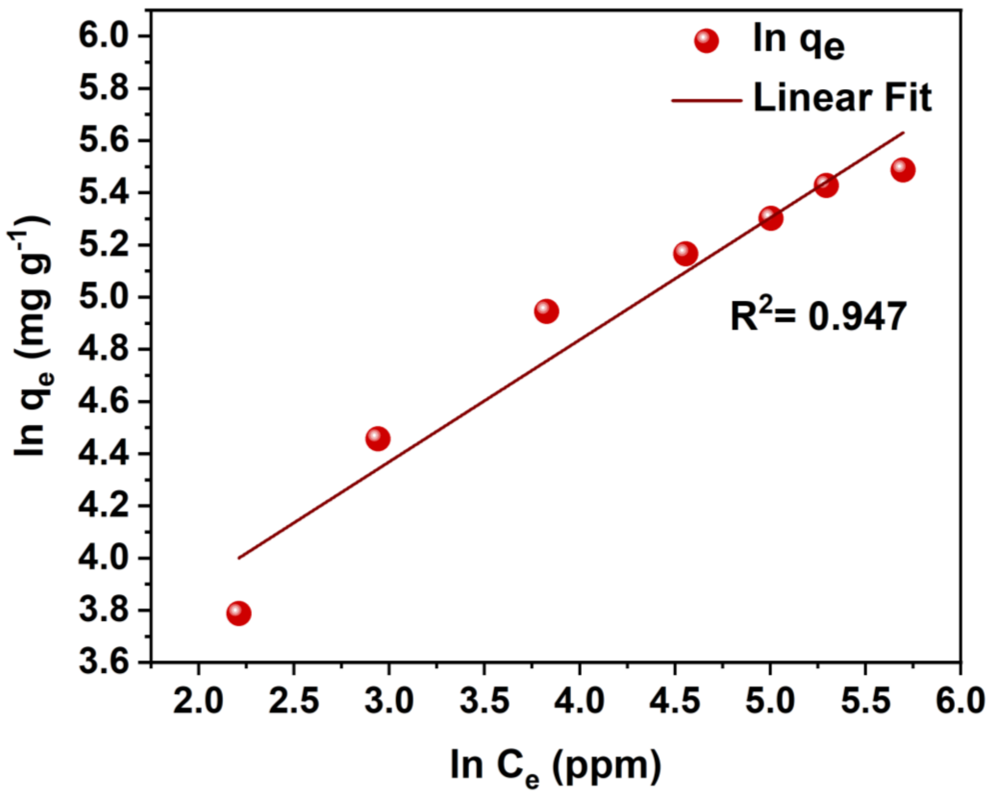

Figure. S8: Freundlich isothermal model fitting for Au^{3+} removal.

Zeta potential of TTASDFP subjected to different Au^{3+} concentrations

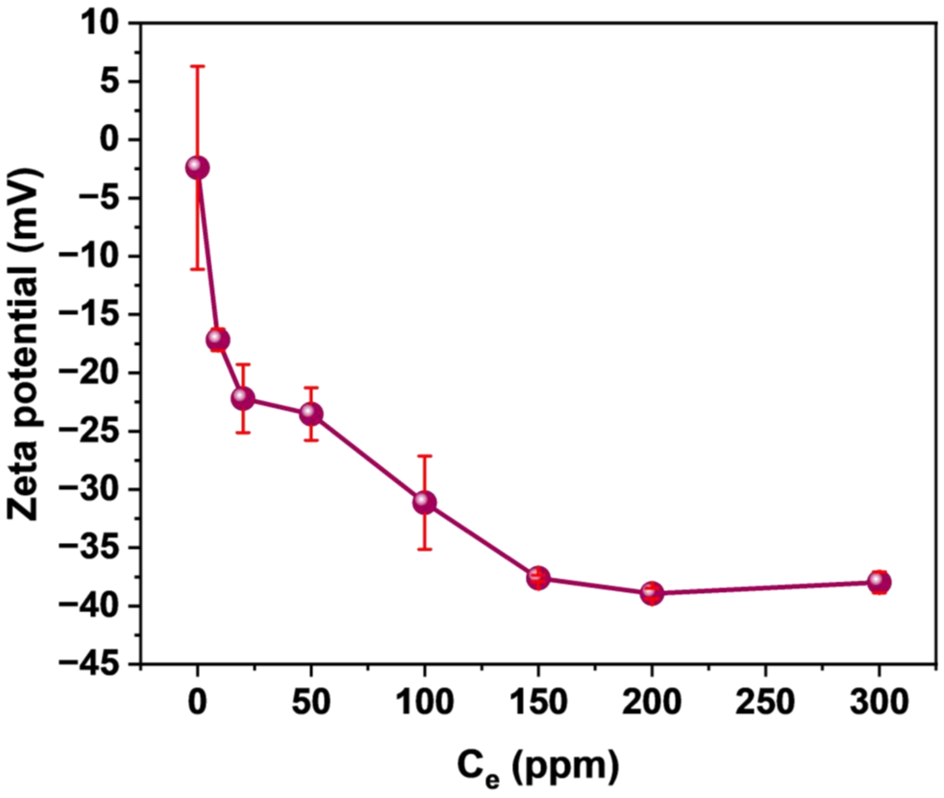

Figure. S9: Zeta potential of TTASDFP with different Au^{3+} adsorption concentrations.

Elemental mapping of TTASDFP with different Au^{3+} adsorption concentrations.

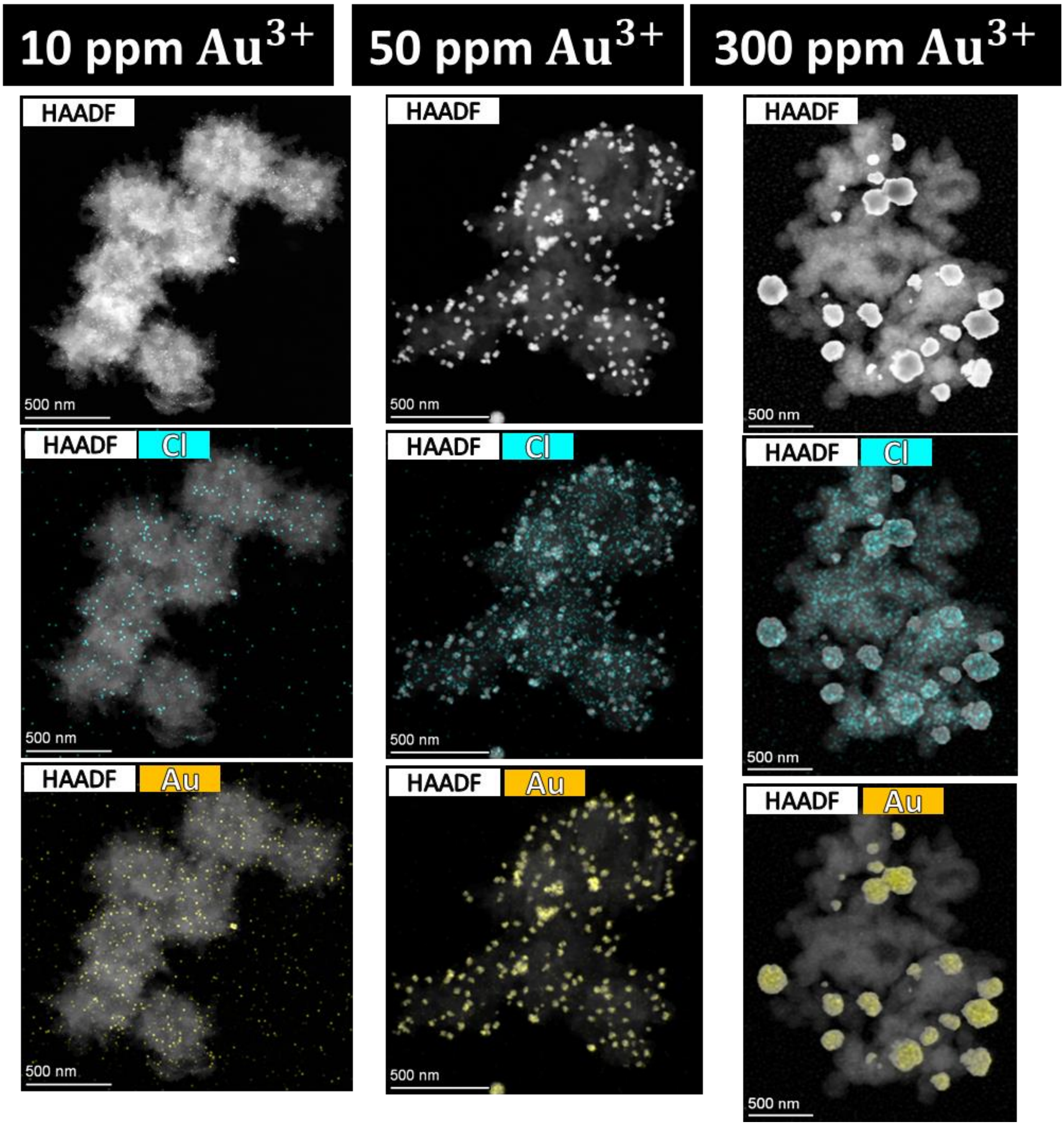

Figure. S10: Elemental mapping of Au and Cl in TTASDFP after exposure to 10, 50 and 300 ppm Au^{3+} isothermal adsorption concentrations.

Energy-Dispersive X-ray Spectroscopy (EDS) analysis of TTASDFP

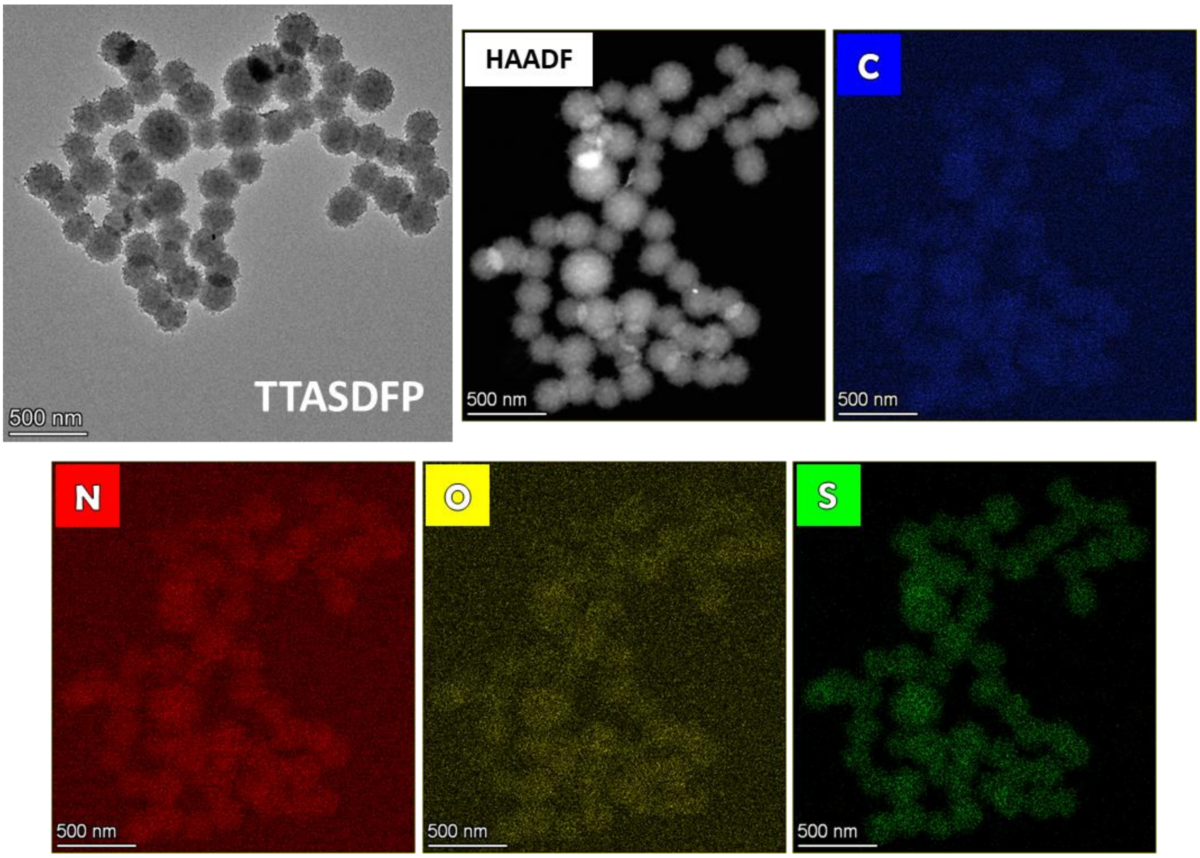

Figure. S11: Energy-dispersive X-ray spectroscopy elemental mapping of TTASDFP

X-ray Photoelectron Spectroscopy (XPS) Survey of TTASDFP and TTASDFP-Au

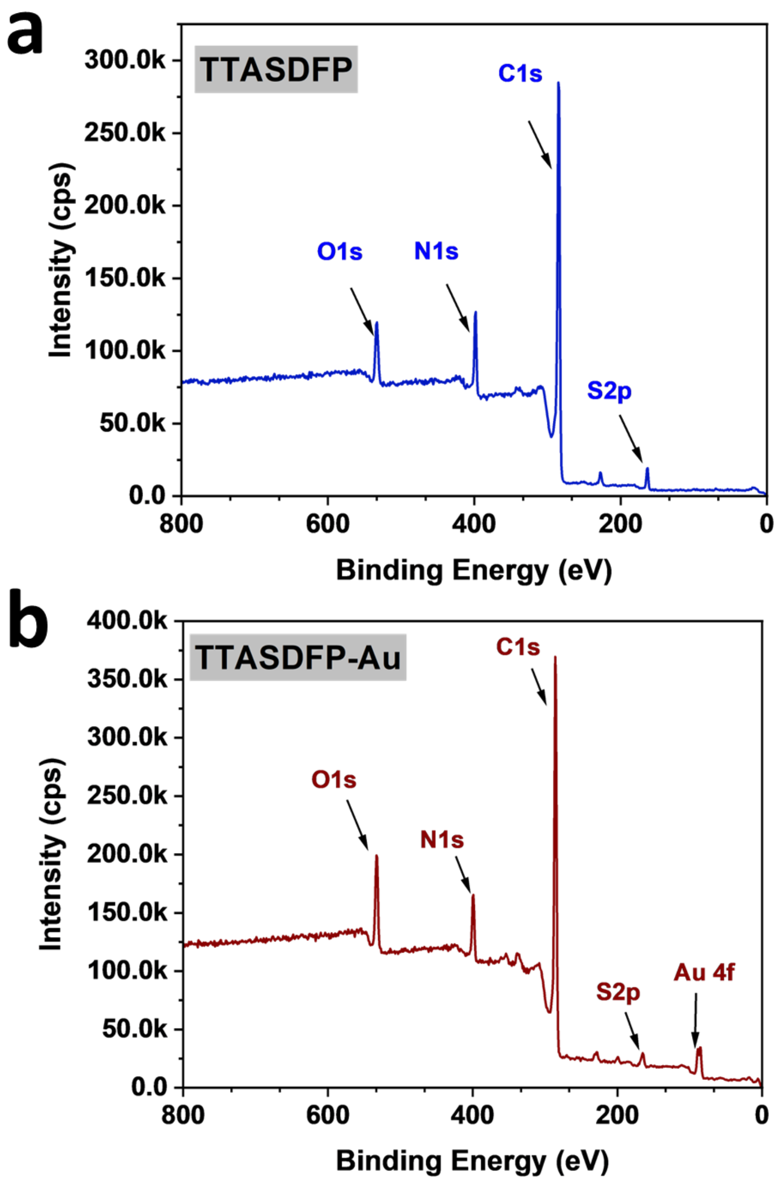

Figure. S12: X-ray Photoelectron Spectroscopy (XPS) survey of a) TTASDFP and b) TTASDFP-Au.

TTASDFP stability under regeneration conditions: PXRD and TEM

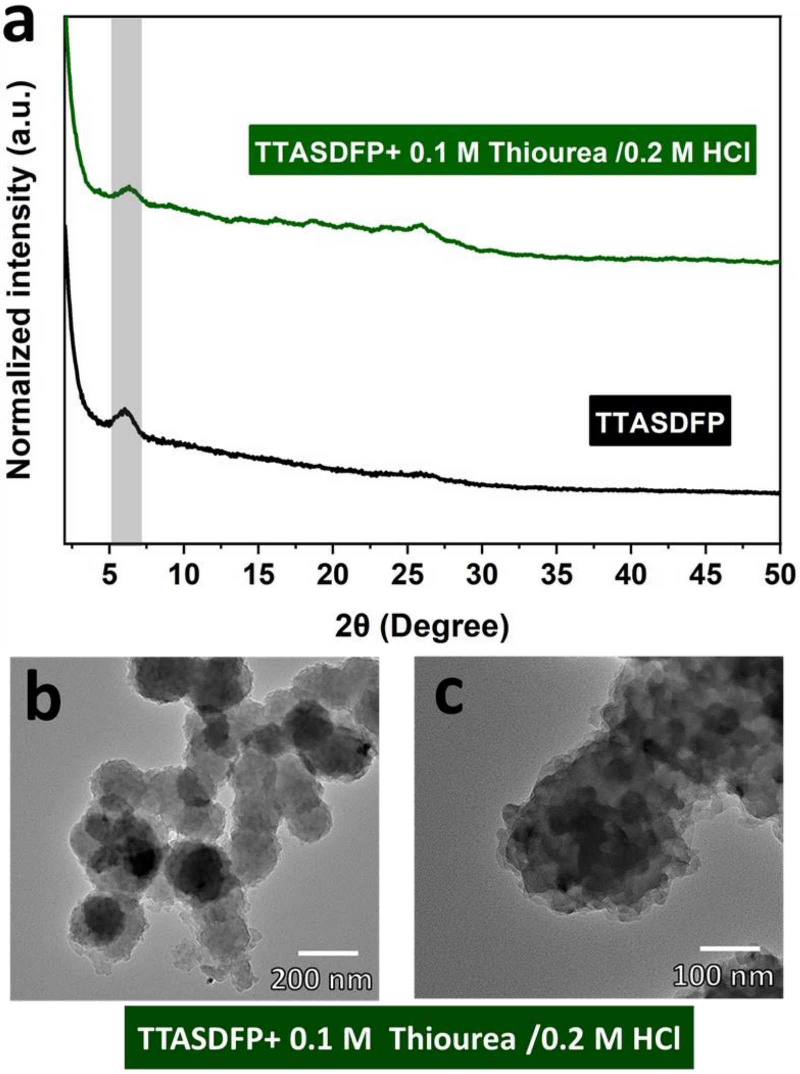

Figure. S13: stacked PXRD patterns of TASDFP before and after exposure to the regeneration solution of 0.1 M thiourea and 0.2 M HCl, b-c) HR-TEM of TTASDFP after treatment with regeneration solution for 5h.

^{13}C CP/MAS NMR analysis of TTASDFP and regenerated TTASDFP

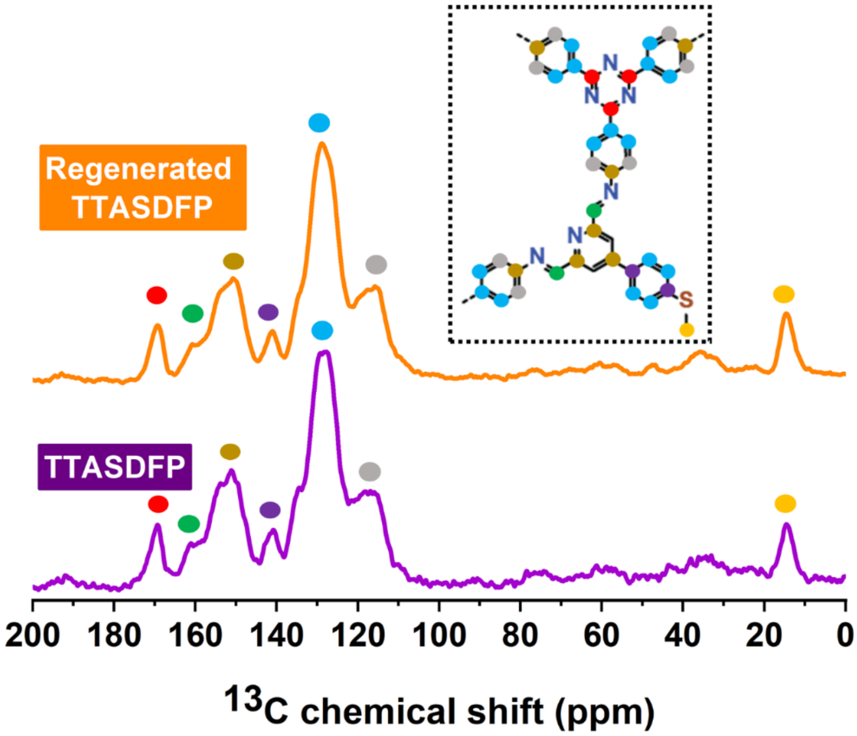

Figure. S14: Stacked ^{13}C CP/MAS NMR spectra of TTASDFP before and after regeneration.

SEM and PXRD analyses of the regenerated TTASDFP

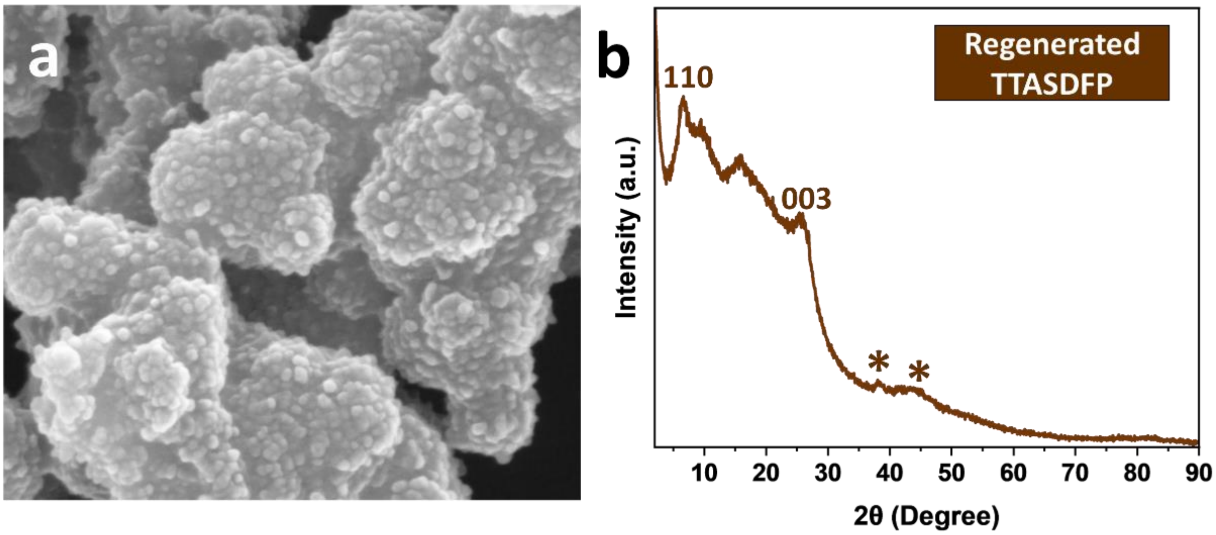

Figure. S15: a) SEM image and b) PXRD pattern of TASDFP-Au after regeneration. The PXRD pattern shows the COF peaks at ~ 6.4° and 26°, in addition to peaks (*) corresponding to gold traces.

Removal of ppb levels of Au^{3+} in the presence of excess NaCl and Cu^{2+}

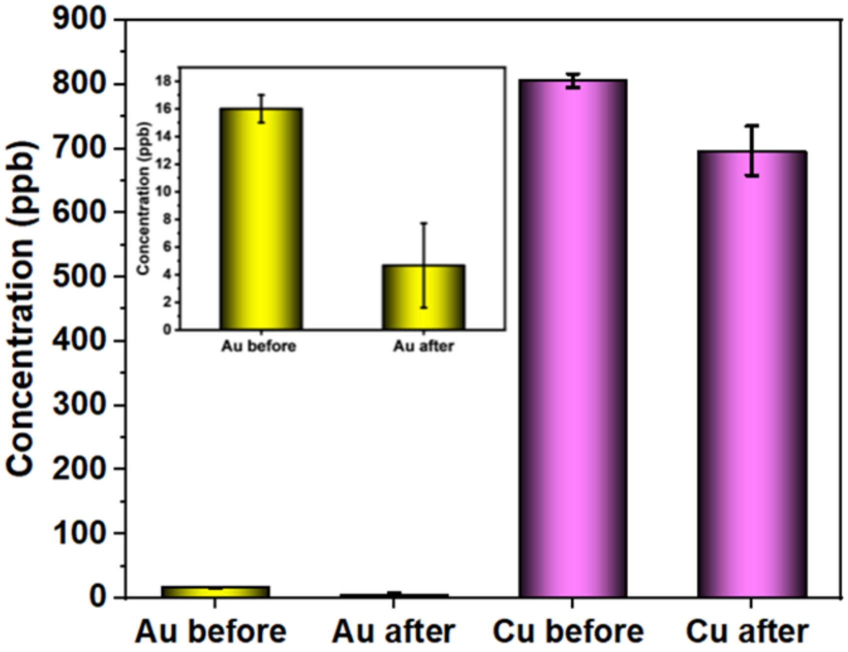

Figure. S16: Concentration of Cu^{2+} andAu^{3+} before and after exposure to TTASDFP in the presence 10,800 ppm NaCl and 806 ppb of $Cu^{2+.}$

Table. S1: Au^{3+} concentrations targeted by previously reported sulfur-based COFs during adsorption studies

Sulfur-based COF	Investigated concentration range of Au^{3+}	Reference
TTR-COF	10 ppm	[5]
SCOFs	10-130 ppm	[6]
TTB-COF	10-400 ppm	[7]
COF-Tp-Tsc	100-1500 ppm	[8]
Tp-BTD COF	25-1200 ppm	[9]
**TTASDFP**	**16 ppb-300 ppm**	**This work**

Table. S2: Comparison of Au^{3+} removal performance for reported adsorbents

Material	Au^{3+} concentration (Kinetic study, ppm)	Equilibrium time	Capacity ($mg{\cdot}g^{-1}$)	Reference
UiO-66-NH_2	400	24 h	650	[10]
MIL-161	200	15 h	446	[11]
Graphene oxide	50	6 h	99	[12]
PAF-1	50	120 min	2629	[13]
SCOFs	10	300 min	100	[6]
COF-Tp-Tsc	300	5 h	4400	[8]
COF-42	200	30 min	219	[14]
COF-HNU26		8 min	1725	
COF-HNU25		8 min	1362	
N^+-PYTA-PATA-COF	25	30 min	1858	[15]
TY-Hz COF	300	2 min	1008	[16]
TTB-COF	10	1 min	560	[7]
JNU-1	396	10 s	1124	[17]
**TTASDFP**	**9**	**30 s**	**245**	**This work**

Table. S3: Au^{3+} adsorption kinetic models for TTASDFP

Kinetic model	Pseudo-first-order			Pseudo-second-order		
**Parameters**	q_e (mg·g^{-1})	K_2 (g·mg^{-1}.min^{-1})	R^2	q_e (mg·g^{-1})	k_2 (g·mg^{-1}·min^{-1})	R^2
**Value**	18.765	0.2314	0.690	18.765	5.6179	1

Table. S4: Au^{3+} adsorption isothermal-models for TTASDFP

Isotherm model	Langmuir			Freundlich		
**Parameters**	q_{max} (mg·g^{-1})	k_L (L·mg^{-1})	R^2	k_f (g^{-1}·mg$^{(1-\frac{1}{n})}$·g^{-1})	n	R^2
**Value**	245	0.0207	0.997	19.42	2.139	0.947

Table. S5: Atomic fractions obtained via STEM at different Au^{3+} concentrations

Au^{3+} concentration (ppm)	Carbon atomic fraction (%)	Gold atomic fraction (%)	Chloride atomic fraction (%)
10	83.41	0.18	0.07
50	96.75	1.07	0.45
300	83.86	5.59	2.84

5. Crystal data of TTASDFP COF model in a cif compatible format

```
data_TTASDFP
_audit_creation_date            2023-12-19
_audit_creation_method          'Materials Studio'
_symmetry_space_group_name_H-M    'P3'
_symmetry_Int_Tables_number       143
_symmetry_cell_setting            trigonal
loop_
_symmetry_equiv_pos_as_xyz
  x,y,z
  -y,x-y,z
  -x+y,-x,z
_cell_length_a                  33.0096
_cell_length_b                  33.0096
_cell_length_c                  10.3970
_cell_angle_alpha                 90.0000
_cell_angle_beta                  90.0000
_cell_angle_gamma                 120.0000
loop_
```

_atom_site_label
_atom_site_type_symbol
_atom_site_fract_x
_atom_site_fract_y
_atom_site_fract_z
_atom_site_U_iso_or_equiv
_atom_site_adp_type
_atom_site_occupancy

H1	H	-0.87120	-1.07706	-0.02560	0.00000	Uiso	1.00
H2	H	-0.94541	-1.07803	-0.02636	0.00000	Uiso	1.00
H3	H	0.13059	-0.92494	-0.03096	0.00000	Uiso	1.00
H4	H	0.20389	-0.92612	-0.03113	0.00000	Uiso	1.00
H5	H	-0.80133	-1.06812	-0.01649	0.00000	Uiso	1.00
H6	H	-0.62754	-1.08072	-0.09246	0.00000	Uiso	1.00
H7	H	-0.75412	-1.29217	-0.06141	0.00000	Uiso	1.00
H8	H	-0.76272	-1.22254	-0.05631	0.00000	Uiso	1.00
H9	H	-0.61233	-1.13527	-0.07547	0.00000	Uiso	1.00
H10	H	-0.60412	-1.20339	-0.08391	0.00000	Uiso	1.00
C11	C	0.04130	-1.00008	-0.03139	0.00000	Uiso	1.00
C12	C	-0.87043	-1.04399	-0.02686	0.00000	Uiso	1.00
C13	C	-0.91313	-1.04476	-0.02744	0.00000	Uiso	1.00
C14	C	0.08615	-1.00025	-0.02959	0.00000	Uiso	1.00
C15	C	0.12920	-0.95841	-0.02998	0.00000	Uiso	1.00
C16	C	0.17122	-0.95907	-0.03035	0.00000	Uiso	1.00
C17	C	0.17207	-1.00111	-0.02823	0.00000	Uiso	1.00
C18	C	-0.77371	-1.03203	-0.02063	0.00000	Uiso	1.00
N19	N	-0.71643	-1.05826	-0.04156	0.00000	Uiso	1.00
C20	C	-0.72489	-1.02215	-0.02550	0.00000	Uiso	1.00
C21	C	0.31295	-0.97609	-0.01576	0.00000	Uiso	1.00
C22	C	0.35918	-0.96727	-0.02533	0.00000	Uiso	1.00
C23	C	0.36600	-1.00545	-0.04606	0.00000	Uiso	1.00
C24	C	-0.67216	-1.05097	-0.05197	0.00000	Uiso	1.00
C25	C	-0.66310	-1.08994	-0.07103	0.00000	Uiso	1.00
C26	C	-0.72302	-1.25796	-0.06569	0.00000	Uiso	1.00
C27	C	-0.72804	-1.21824	-0.06286	0.00000	Uiso	1.00
C28	C	-0.68879	-1.17323	-0.06695	0.00000	Uiso	1.00
C29	C	-0.64404	-1.16879	-0.07438	0.00000	Uiso	1.00
C30	C	-0.63906	-1.20833	-0.07831	0.00000	Uiso	1.00
C31	C	-0.67834	-1.25341	-0.07241	0.00000	Uiso	1.00
C32	C	-0.67232	-1.29510	-0.07199	0.00000	Uiso	1.00
N33	N	-1.00006	-1.04106	-0.03135	0.00000	Uiso	1.00
N34	N	0.21716	-0.99802	-0.02703	0.00000	Uiso	1.00
N35	N	-0.69572	-1.13342	-0.05839	0.00000	Uiso	1.00
N36	N	-0.66109	-1.37127	-0.07197	0.00000	Uiso	1.00
H37	H	0.40383	-1.06573	0.60839	0.00000	Uiso	1.00
H38	H	0.25979	-1.27692	0.60568	0.00000	Uiso	1.00
H39	H	0.26104	-1.20238	0.60952	0.00000	Uiso	1.00
H40	H	0.41183	-1.12849	0.60594	0.00000	Uiso	1.00
H41	H	0.41095	-1.20275	0.60414	0.00000	Uiso	1.00
C42	C	0.31030	-1.04565	0.62163	0.00000	Uiso	1.00
C43	C	0.30002	-1.00951	0.63896	0.00000	Uiso	1.00
C44	C	-1.66232	-0.96379	0.65441	0.00000	Uiso	1.00
C45	C	-1.61612	-0.95482	0.64981	0.00000	Uiso	1.00
N46	N	-1.60763	-0.99094	0.63493	0.00000	Uiso	1.00
C47	C	0.35692	-1.03600	0.62213	0.00000	Uiso	1.00
C48	C	0.36767	-1.07433	0.61132	0.00000	Uiso	1.00
C49	C	0.29328	-1.24487	0.60539	0.00000	Uiso	1.00
C50	C	0.29387	-1.20231	0.60729	0.00000	Uiso	1.00
C51	C	0.33613	-1.15981	0.60662	0.00000	Uiso	1.00
C52	C	0.37843	-1.16047	0.60534	0.00000	Uiso	1.00

C53	C	0.37792	-1.20323	0.60461	0.00000	Uiso	1.00
C54	C	0.33528	-1.24598	0.60346	0.00000	Uiso	1.00
C55	C	0.33431	-1.29148	0.60122	0.00000	Uiso	1.00
N56	N	-1.32827	-0.70491	0.67492	0.00000	Uiso	1.00
N57	N	0.33370	-1.11754	0.61077	0.00000	Uiso	1.00
N58	N	-0.66761	-0.37481	0.60126	0.00000	Uiso	1.00
H59	H	-1.53169	-0.80985	0.64088	0.00000	Uiso	1.00
H60	H	-0.46331	-1.73326	0.65011	0.00000	Uiso	1.00
H61	H	-1.37578	-0.79532	0.69185	0.00000	Uiso	1.00
H62	H	-1.44548	-0.87335	0.68425	0.00000	Uiso	1.00
H63	H	-1.58718	-0.87937	0.67101	0.00000	Uiso	1.00
H64	H	-1.79570	-0.92324	0.28546	0.00000	Uiso	1.00
H65	H	-0.86954	-1.92360	0.28525	0.00000	Uiso	1.00
H66	H	-0.94456	-1.07464	0.30204	0.00000	Uiso	1.00
H67	H	-0.87020	-1.07398	0.30166	0.00000	Uiso	1.00
H68	H	-1.73359	-0.93126	0.29370	0.00000	Uiso	1.00
H69	H	-1.54681	-0.91952	0.36068	0.00000	Uiso	1.00
H70	H	-0.46202	-1.70815	0.33540	0.00000	Uiso	1.00
H71	H	-1.54019	-0.77777	0.33053	0.00000	Uiso	1.00
H72	H	-1.47706	-0.86511	0.32961	0.00000	Uiso	1.00
H73	H	-1.40047	-0.79688	0.33634	0.00000	Uiso	1.00
C74	C	-1.28964	-0.66154	0.67501	0.00000	Uiso	1.00
C75	C	-1.49840	-0.80795	0.65454	0.00000	Uiso	1.00
C76	C	-1.45875	-0.76342	0.65899	0.00000	Uiso	1.00
C77	C	-1.41391	-0.75789	0.67257	0.00000	Uiso	1.00
C78	C	-1.40977	-0.79819	0.68171	0.00000	Uiso	1.00
C79	C	-1.44954	-0.84284	0.67751	0.00000	Uiso	1.00
C80	C	-1.49427	-0.84839	0.66434	0.00000	Uiso	1.00
C81	C	-1.57763	-0.90605	0.66051	0.00000	Uiso	1.00
C82	C	-0.95841	-0.99957	0.29498	0.00000	Uiso	1.00
C83	C	-1.82772	-0.95655	0.28937	0.00000	Uiso	1.00
C84	C	-1.87028	-0.95674	0.28906	0.00000	Uiso	1.00
C85	C	-0.91317	-0.99916	0.29375	0.00000	Uiso	1.00
C86	C	-0.91237	-1.04131	0.29826	0.00000	Uiso	1.00
C87	C	-0.86992	-1.04105	0.29814	0.00000	Uiso	1.00
C88	C	-0.82729	-0.99896	0.29344	0.00000	Uiso	1.00
C89	C	-1.74191	-0.96734	0.29319	0.00000	Uiso	1.00
N90	N	-1.65829	-0.94152	0.31338	0.00000	Uiso	1.00
C91	C	-1.70307	-0.97742	0.29903	0.00000	Uiso	1.00
C92	C	-0.71174	-1.02364	0.29109	0.00000	Uiso	1.00
C93	C	-0.67466	-1.03290	0.30020	0.00000	Uiso	1.00
C94	C	-0.62931	-0.99494	0.31768	0.00000	Uiso	1.00
C95	C	-1.62148	-0.94919	0.32272	0.00000	Uiso	1.00
C96	C	-1.57320	-0.91033	0.33924	0.00000	Uiso	1.00
C97	C	-1.46503	-0.74235	0.33518	0.00000	Uiso	1.00
C98	C	-1.50977	-0.78208	0.33261	0.00000	Uiso	1.00
C99	C	-1.51547	-0.82708	0.33222	0.00000	Uiso	1.00
C100	C	-1.47510	-0.83153	0.33324	0.00000	Uiso	1.00
C101	C	-1.43053	-0.79197	0.33579	0.00000	Uiso	1.00
C102	C	-1.42482	-0.74686	0.33656	0.00000	Uiso	1.00
C103	C	-1.37716	-0.70504	0.33763	0.00000	Uiso	1.00
N104	N	-1.53397	-0.89522	0.65768	0.00000	Uiso	1.00
N105	N	-0.95919	-1.95878	0.29495	0.00000	Uiso	1.00
N106	N	-0.78503	-1.00153	0.29354	0.00000	Uiso	1.00
N107	N	-1.56227	-0.86687	0.32558	0.00000	Uiso	1.00
N108	N	-0.28990	-1.62861	0.33764	0.00000	Uiso	1.00
H109	H	0.30623	-0.94769	0.00172	0.00000	Uiso	1.00
C110	C	0.40027	-0.91871	-0.01669	0.00000	Uiso	1.00
H111	H	0.40082	-1.00012	-0.05979	0.00000	Uiso	1.00
H112	H	0.28242	-1.08113	0.60617	0.00000	Uiso	1.00

C113	*C*	*0.25045*	*-1.01941*	*0.64092*	*0.00000*	*Uiso*	*1.00*
H114	*H*	*-1.66843*	*-0.93499*	*0.67203*	*0.00000*	*Uiso*	*1.00*
H115	*H*	*-0.74703*	*-1.05195*	*0.27512*	*0.00000*	*Uiso*	*1.00*
C116	*C*	*-0.68306*	*-1.08179*	*0.29299*	*0.00000*	*Uiso*	*1.00*
H117	*H*	*-0.59998*	*-1.00066*	*0.32956*	*0.00000*	*Uiso*	*1.00*
C118	*C*	*1.64486*	*1.55591*	*1.02581*	*0.00000*	*Uiso*	*1.00*
C119	*C*	*1.65069*	*1.51687*	*1.02701*	*0.00000*	*Uiso*	*1.00*
C120	*C*	*1.69306*	*1.52047*	*0.99008*	*0.00000*	*Uiso*	*1.00*
C121	*C*	*1.72967*	*1.56416*	*0.94910*	*0.00000*	*Uiso*	*1.00*
C122	*C*	*1.72363*	*1.60322*	*0.94542*	*0.00000*	*Uiso*	*1.00*
S123	*S*	*1.69649*	*1.46694*	*0.99668*	*0.00000*	*Uiso*	*1.00*
C124	*C*	*1.75940*	*1.49051*	*0.97333*	*0.00000*	*Uiso*	*1.00*
H125	*H*	*1.61200*	*1.55153*	*1.06027*	*0.00000*	*Uiso*	*1.00*
H126	*H*	*1.62205*	*1.48358*	*1.05887*	*0.00000*	*Uiso*	*1.00*
H127	*H*	*1.76273*	*1.56869*	*0.91694*	*0.00000*	*Uiso*	*1.00*
H128	*H*	*1.75199*	*1.63567*	*0.90843*	*0.00000*	*Uiso*	*1.00*
H129	*H*	*1.77951*	*1.51754*	*1.04704*	*0.00000*	*Uiso*	*1.00*
H130	*H*	*1.76990*	*1.50505*	*0.87496*	*0.00000*	*Uiso*	*1.00*
H131	*H*	*1.76708*	*1.46158*	*0.98425*	*0.00000*	*Uiso*	*1.00*
C132	*C*	*2.60560*	*1.72566*	*0.33443*	*0.00000*	*Uiso*	*1.00*
C133	*C*	*2.56772*	*1.73338*	*0.32656*	*0.00000*	*Uiso*	*1.00*
C134	*C*	*2.52427*	*1.69853*	*0.28073*	*0.00000*	*Uiso*	*1.00*
C135	*C*	*2.51952*	*1.65581*	*0.24004*	*0.00000*	*Uiso*	*1.00*
C136	*C*	*2.55762*	*1.64836*	*0.24509*	*0.00000*	*Uiso*	*1.00*
S137	*S*	*2.47703*	*1.71210*	*0.27378*	*0.00000*	*Uiso*	*1.00*
C138	*C*	*2.42605*	*1.65281*	*0.27094*	*0.00000*	*Uiso*	*1.00*
H139	*H*	*2.63796*	*1.75298*	*0.37512*	*0.00000*	*Uiso*	*1.00*
H140	*H*	*2.57206*	*1.76652*	*0.35864*	*0.00000*	*Uiso*	*1.00*
H141	*H*	*2.48712*	*1.62835*	*0.20079*	*0.00000*	*Uiso*	*1.00*
H142	*H*	*2.55289*	*1.61565*	*0.20806*	*0.00000*	*Uiso*	*1.00*
H143	*H*	*2.42287*	*1.63606*	*0.17674*	*0.00000*	*Uiso*	*1.00*
H144	*H*	*2.39400*	*1.65505*	*0.28706*	*0.00000*	*Uiso*	*1.00*
H145	*H*	*2.42885*	*1.63107*	*0.34745*	*0.00000*	*Uiso*	*1.00*
C146	*C*	*2.23948*	*1.01532*	*0.60673*	*0.00000*	*Uiso*	*1.00*
C147	*C*	*2.19329*	*1.00611*	*0.60810*	*0.00000*	*Uiso*	*1.00*
C148	*C*	*2.15655*	*0.96161*	*0.64212*	*0.00000*	*Uiso*	*1.00*
C149	*C*	*2.16715*	*0.92697*	*0.67614*	*0.00000*	*Uiso*	*1.00*
C150	*C*	*2.21337*	*0.93628*	*0.67686*	*0.00000*	*Uiso*	*1.00*
S151	*S*	*2.09487*	*0.94533*	*0.64142*	*0.00000*	*Uiso*	*1.00*
C152	*C*	*2.09996*	*1.00327*	*0.63798*	*0.00000*	*Uiso*	*1.00*
H153	*H*	*2.26625*	*1.04954*	*0.57492*	*0.00000*	*Uiso*	*1.00*
H154	*H*	*2.18701*	*1.03382*	*0.57881*	*0.00000*	*Uiso*	*1.00*
H155	*H*	*2.13940*	*0.89255*	*0.70387*	*0.00000*	*Uiso*	*1.00*
H156	*H*	*2.21971*	*0.90876*	*0.70861*	*0.00000*	*Uiso*	*1.00*
H157	*H*	*2.06479*	*0.99914*	*0.65234*	*0.00000*	*Uiso*	*1.00*
H158	*H*	*2.11357*	*1.02037*	*0.54424*	*0.00000*	*Uiso*	*1.00*
H159	*H*	*2.12349*	*1.02565*	*0.71528*	*0.00000*	*Uiso*	*1.00*

References:

1. Halder, A., et al., Decoding the Morphological Diversity in Two Dimensional Crystalline Porous Polymers by Core Planarity Modulation. *Angew Chem Int Edit*. **2016**, 55, 7806-7810.

2. Das, G., et al., Hydrophobicity Tuning in Isostructural Urchin-Shaped Covalent Organic Framework Nanoparticles by Pore Surface Engineering for Oil-Water Separation. *Acs Appl Nano Mater*. **2022**, 5, 13745–13751.
3. Ateia, M., D.E. Helbling, and W.R. Dichtel, Best Practices for Evaluating New Materials as Adsorbents for Water Treatment. *Acs Mater Lett*. **2020**, 2, 1532-1544.
4. Ji, W., et al., Removal of GenX and Perfluorinated Alkyl Substances from Water by Amine-Functionalized Covalent Organic Frameworks. *J Am Chem Soc*. **2018**, 140, 12677-12681.
5. Li, L.Y., et al., Thioether-Functionalized 2D Covalent Organic Framework Featuring Specific Affinity to Au for Photocatalytic Hydrogen Production from Seawater. *Acs Sustain Chem Eng*. **2019**, 7, 18574-18581.
6. Liu, C., et al., A Facile Approach to Sulfur-Rich Covalent Organic Frameworks for Selective Recovery of Trace Gold. *Macromol Mater Eng*. **2022**, 307, 2100761.
7. Zhou, Z.M., et al., A covalent organic framework bearing thioether pendant arms for selective detection and recovery of Au from ultra-low concentration aqueous solution. *Chem Commun*. **2018**, 54, 9977-9980.
8. Zhang, L., et al., Covalent organic frameworks constructed by flexible alkyl amines for efficient gold recovery from leaching solution of e-waste. *Chem Eng J*. **2021**, 426, 131865.
9. Yang, S.X., et al., Covalent Organic Framework Isomers for Photoenhanced Gold Recovery from E-Waste with High Efficiency and Selectivity. *Acs Sustain Chem Eng*. **2022**, 10, 9719-9731.
10. Chang, Z.Y., et al., Selective and efficient adsorption of Au (III) in aqueous solution by Zr-based metal-organic frameworks (MOFs): An unconventional way for gold recycling. *J Hazard Mater*. **2020**, 391, 122175.
11. Hu, G.Y., et al., MIL-161 Metal-Organic Framework for Efficient Au(III) Recovery from Secondary Resources: Performance, Mechanism, and DFT Calculations. *Molecules*. **2023**, 28, 5459.
12. Liu, L., et al., Adsorption of Au(III), Pd(II), and Pt(IV) from Aqueous Solution onto Graphene Oxide. *J Chem Eng Data*. **2013**, 58, 209-216.
13. Ma, T.T., et al., Efficient Gold Recovery from E-Waste via a Chelate-Containing Porous Aromatic Framework. *Acs Appl Mater Inter*. **2020**, 12, 30474-30482.
14. Qiu, J.K., et al., Porous Covalent Organic Framework Based Hydrogen-Bond Nanotrap for the Precise Recognition and Separation of Gold. *Angew Chem Int Edit*. **2023**, e202300459.
15. Liu, M., et al., Modulating Skeletons of Covalent Organic Framework for High-Efficiency Gold Recovery. *Angew Chem Int Ed Engl*. **2023**, e202317015.
16. Zhang, L., et al., A 2D mesoporous hydrazone covalent organic framework for selective detection and ultrafast recovery of Au(III) from electronic waste. *Chem Eng J*. **2023**, 454, 140212.
17. Qian, H.L., et al., Irreversible Amide-Linked Covalent Organic Framework for Selective and Ultrafast Gold Recovery. *Angew Chem Int Edit*. **2020**, 59, 17607-17613.
